# Mucormycosis Amid COVID-19 Crisis: Pathogenesis, Diagnosis, and Novel Treatment Strategies to Combat the Spread

**DOI:** 10.3389/fmicb.2021.794176

**Published:** 2022-01-04

**Authors:** Shreya Dogra, Akanksha Arora, Aashni Aggarwal, Gautam Passi, Akanksha Sharma, Gurpal Singh, Ravi P. Barnwal

**Affiliations:** ^1^Department of Biophysics, Panjab University, Chandigarh, India; ^2^University Institute of Pharmaceutical Sciences, Panjab University, Chandigarh, India

**Keywords:** SARS-CoV-2, mucormycosis, black fungus, amphotericin B, antifungal drugs

## Abstract

The havoc unleashed by COVID-19 pandemic has paved way for secondary ominous fungal infections like Mucormycosis. It is caused by a class of opportunistic pathogens from the order Mucorales. Fatality rates due to this contagious infection are extremely high. Numerous clinical manifestations result in damage to multiple organs subject to the patient’s underlying condition. Lack of a proper detection method and reliable treatment has made the management of this infection troublesome. Several reports studying the behavior pattern of Mucorales inside the host by modulation of its defense mechanisms have helped in understanding the pathogenesis of this angio-invasive infection. Many recent advances in diagnosis and treatment of this fungal infection have not been much beneficial. Therefore, there is a need to foster more viable strategies. This article summarizes current and imminent approaches that could aid effective management of these secondary infections in these times of global pandemic. It is foreseen that the development of newer antifungal drugs, antimicrobial peptides, and nanotechnology-based approaches for drug delivery would help combat this infection and curb its spread.

## Introduction

There is a surge of secondary infections amid the global COVID-19 pandemic. Mucormycosis has been reported in many countries as one of the COVID-19 related comorbidities. The region affected by the fungi appears black, which is why the disease is commonly referred as “black fungus.” The organs commonly targeted by this disease include nose, brain, eyes, sinuses, lungs, Gastrointestinal (GI) tract, skin, and kidneys. Some of the noticeable symptoms of this infection include swollen eyes, runny nose, blurred vision, and facial swelling (Petrikkos et al., 2012). One of the most remarkable signs of this disease is tissue necrosis, which is often a result of vascular thrombosis. It is a non-contagious, angio-invasive infection caused by members of the order Mucorales belonging to the kingdom fungi. It was earlier known by the name zygomycosis as the species were believed to belong to phylum Zygomycota, but now the agents causing mucormycosis are categorized in a new phylum Glomeromycota under subphylum mucoromycota; the order Mucorales falls under the subdivision mucoromycotina (Kwon-Chung, 2012). The fungi mainly responsible for this infection include Mucor and Rhizopus along with *Cunninghamella* sp., *Saksenaea* sp., *Lichtheimia* sp., *Apophysomyces* sp., *Rhizomucor* sp., and *Cokeromyces* sp. (Roden et al., 2005). The spores are dispersed in the surrounding air and can be easily transmitted by the inhalation of droplets (Richardson, 2009). The earliest description of this disease dates back to 1876 when the lungs of a cancer patient were found to harbor sporangia and fungal hyphae with hemorrhagic infarct. Almost a decade later, in the year 1885, the first incidence of mycosis mucorina, also known as disseminated mucormycosis was reported (Paltauf, 1885). The overall fatality rate of this infection from across the globe is estimated to be 46% (Werthman-Ehrenreich, 2021). These fungal species are the second most prevalent type of molds following *Aspergillus*, which is also an opportunist secondary pathogen (Slavin et al., 2015).

Mucormycosis is an uncommon infection more prevalent in regions affected by natural calamities (Dannaoui, 2002). However, owing to the global pandemic, around 1.7 people in every 1,000,000 are contracting this disease (Bouza et al., 2006). As per the World Health Organization (WHO), the pervasiveness of this infection in India is 140 per million populations (Chakrabarti and Dhaliwal, 2013). Monoclonal antibodies like tocilizumab and itolizumab used for the treatment of COVID-19 cause immune suppression that makes patients vulnerable to these ubiquitous fungi.

The mucorales easily multiply when they encounter a favorable environment in the immunocompromised individuals. The pathogenesis of infection is majorly due to the inbuilt host defense mechanism. The risk factors that make a person more susceptible to this disease include diabetes, COVID-19, surgeries, hematological malignancies (HM), uptake of corticosteroids, hospital acquired etc. Complications associated with this infection include blindness and thrombosis. It is advisable to take proper preventive measures and maintain good personal hygiene to reduce disease occurrence. Diagnosis involves the use of many new and conventional methods like biopsy, CT scans, and PCR based methods. The treatment options available are not much effective due to resemblance of the drug targets in the pathogen with the host (Riley et al., 2016), but antifungal therapy and surgical removal of the affected region are suggested.

The novel strategies of treatment based on targeted drug deliveries of the antifungal drugs have exhibited exceptional fungicidal activity that can be exploited for treatment. The main aim of this review is to provide a single platform that incorporates details about pathogenesis, epidemiology, clinical manifestations, underlying factors, diagnostic methods, and conventional and novel treatment approaches for better management of this invasive infection and reduce the upsurge of black fungus cases.

## Morphology of Mucorales

Fungi categorized under Order Mucorales are ubiquitous in nature and serve as an integral part of human environment, colonizing the wet organic materials, fermenting various food and drinks, production of cheese, and causative agent for several life-threatening infections.

Mucorales reproduce either by sexual or asexual means; sexual reproduction involves production of zygospores that are pigmented and thick walled. Fusion/blending of two separated ends of hyphae including the blending of nuclei and cytoplasm of zygospores result in the formation of zygote that develops into a mature fungus. On the other hand, asexual reproduction includes the formation of endogenously produced uni-celled spores, called sporangiospores. In case of sporangiospores, the formation of cell wall is without the association of pre-existing cell walls. Columellae have been reported from two orders: well-defined columellae in Mucorales and inconspicuous columellae in Umbelopsidales. It has been observed in histopathological studies that Entomophthoromycoses and Mucormycoses are identical owing to non or rarely septate, belt like, and broad hyphae. Moreover, these can be differentiated using hematoxylin and eosin (H&E) staining, wherein eosinophilic sleeves enclose the Entomophthorales hyphae only. According to a study by Jeong et al. (2019), Mucor is a part of the main genera causing mucormycosis. The main characteristics of the genus Rhizopus includes: sporangia possessing apophysis and pigmented, unbranched sporangiophores originating either in whorls or singly. Rapidly growing colonies with hyphae, which form arched stolons along with rhizoids, are the primary feature of Absidia. Other characteristics include: pear shaped sporangia, sporangium with apophysis below, and presence of stolons from where sporangiophores originate. Sporangiophores are normally upright hyphae, which can be simple, slightly branched and septate, usually in fascicles on common base.

## Pathogenesis

### Virulence Factors

Virulence strategies of the fungal pathogen influence its morphology, which further directs the pathogenesis mechanism (Min et al., 2020). Interaction of fungi with the immune system is influenced by the cell morphology (Erwig and Gow, 2016). *Rhizopus oryzae* is normally present in its sporangiophore form and changes to coenocytic hyphae in the host cell. Mucorales compromise the immune system by shape shifting in the host cells to form large aggregates/clusters of cells or long hyphae, which cannot be phagocytized by leukocytes as developing hyphae rupture out of macrophages. Few shape shifting fungi form interconnected hyphae (mycelium) that help in nutrient sharing and hence, promote growth (Min et al., 2020). Genetic modifications over the course of time have made the pathogen competent enough to take nutrition from host, develop resistance to antifungal drugs for survival against host immunity and in adverse conditions by maintaining high growth and metabolism, synthesizing virulence factors, hastened cell wall synthesis, and immense iron uptake (Ibrahim et al., 2008; Lamaris et al., 2009; Lewis et al., 2012).

**Figure 1:**
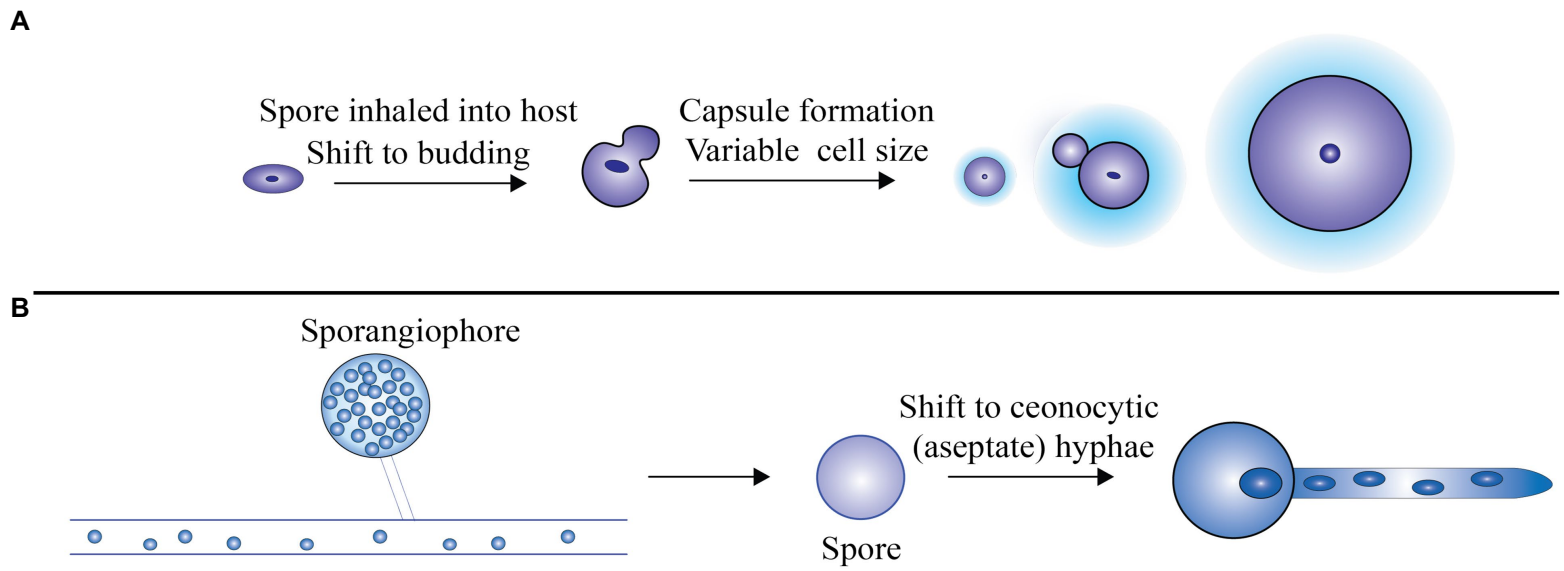
**(A)** The spores revert back to the budding mode of replication after being inhaled by the host. For protecting the cells against stress and unfavorable environment, a thick polysaccharide capsule is formed and also the formation of large cells that are immune to phagocytosis, **(B)** *Rhizopus oryzae* grows as coenocytic (aseptate) hyphae, which produce few septa in the host. Adapted from Min et al. (2020).

Virulence of Mucorales further depends on the secretion of lytic enzymes encoded by a number of genes and also metabolites like mycotoxins and alkaloids that promote intrusion of tissue and suppress host immune response (Ribes et al., 2000; Challa, 2019). Mucorales spores can enter the host cell *via* inhalation, ingestion of contaminated food, or through local inoculation. Figure 1 diagrammatically represents the transformation of spores into coenocytic (aseptate) hyphae when transmitted to the host tissue.

### Defense Against Innate Immunity

Mucorales spores bind extracellular matrix (ECM) proteins collagen IV and laminin in basement membrane. They dismantle the stroma and invade the host cell by synthesizing glycosidic enzymes, proteases, and subtilases (Schoen et al., 2002; Spreer et al., 2006). After overcoming physical barriers of the skin and mucosal lining, the second line of defense, i.e., cellular response is activated which comprises of macrophages, mononuclear cells, neutrophils, and dendritic cells (Ibrahim and Voelz, 2017). The inhaled asexual sporangiospores that are omnipresent in the environment are phagocytosed by the macrophages. Escape of spores from the body’s defense system leads to their development into hyphae, further promoting chemotaxis of neutrophils, followed by phagocytosis and killing of hyphae. This further includes elimination of hyphae and spores by neutrophils *via* oxidative cytotoxicity. These neutrophils produce perforins, various reactive oxygen metabolites, enzymes, and cationic peptides. Additionally, they also synthesize pro-inflammatory cytokines, such as interleukin-1b (IL-1b), tumor necrosis factor (TNF-α), and interferon-gamma (INF-γ), which are further involved in recruitment and activation of other inflammatory cells. The fungal pathogen has Pathogen Associated Molecular Patterns (PAMPs) present on its surface to which the recognition receptors like Toll-like receptors (TLRs) at the phagocytes bind and activate the intracellular signaling and inflammatory process (Roilides et al., 2012).

Platelets contain three types of cytoplasmic granules: dense granules, which store mediators like adenosine nucleotide diphosphate and serotonin; alpha granules, associated with coagulation and adhesion; and lysosomal granules, consisting of lysosomal enzymes (Fitzgerald et al., 2006). Because of its characteristic antifungal and antimicrobial properties, platelets have crucial role in the host immune response by secreting granules, which consist of anti-inflammatory and pro-inflammatory cytokines, like transforming growth factor β, chemokines, and thrombocidins with fungicidal properties. Attachment to Mucorales spore and hyphae activates the platelets that initiate clot formation and aggregation, further inhibiting hematogenous dissemination of fungi (Perkhofer et al., 2009; Ibrahim and Voelz, 2017). Natural killer (NK) cells prompt cytotoxic effects by producing chemokines and cytokines like granulocyte-macrophage colony-stimulating factor (GMCSF), IFN-γ, and TNF-α, thus exerting their impact on other immune cells and ultimately destroying fungal hyphae (Perkhofer et al., 2009).

### CotH Proteins

By the expression of spore coat homolog (CotH) proteins, Mucorales bind with the monolayer of endothelial cells, which comprises cellular lining of blood vessels, and is in direct contact with the body (Spreer et al., 2006). These are exclusive to Mucorales and the number of copies expressed influence its virulence. Generally, disease causing species like *Rhizopus* sp., *Mucor* sp., and *Lichtheimia* sp. express 3–7 copies of CotH genes compared to those which cause disease less frequently like *Apophysomyces* sp., *Cunninghamella* sp., *Saksenaea* sp., and *Syncephalastrum* sp. which express 1–2 copies of CotH genes (Chibucos et al., 2016). CotH, with a short half-life of 4–5 h is a protein kinase belonging to the spore coating protein family. It is needed for assembling proteins in the inner spore coat layer. During sporulation, CotH activity is regulated by autophosphorylation using ATP. Its concentration decreases rapidly after the transcription of structural gene is deactivated. As per recent research, it has been suggested to be an important component of spore germination in many bacteria (Gebremariam et al., 2014).

The CotH proteins act as fungal ligands to receptor Glucose Regulatory Protein (GRP) 78. Germlings of *R. oryzae* adhere to these proteins rather than spores while invading the host (Roilides et al., 2014; Challa, 2019). GRP 78 (also known as HSPA5/BiP) is a cellular heat shock protein present in endoplasmic reticulum. The primary role of GRP 78 is as a chaperone associated with many cellular processes like assembly and folding of protein, labeling of misfolded proteins for proteasome degradation, sensor for endoplasmic reticulum stress, and maintaining calcium homeostasis (Ibrahim et al., 2008). Fungal ligand CotH binds with GRP 78 receptor present on the endothelial cell, which aids in its endocytosis leading to its damage. This adherence is supported by hyperglycemia and iron in acidic pH. Some toxic secondary metabolites secreted by Mucorales also promote endothelial damage (Challa, 2019).

### Iron Uptake

Iron is required in the host cell as it facilitates differentiation of lymphocyte and macrophages, acts as anti-microbial immune effector and is involved in metabolism of immune cells. Iron assumes a crucial role in the development of fungal cell wall, as in iron deprived conditions, fungi undergoes apoptosis (Ibrahim and Kontoyiannis, 2013).

**Figure 2:**
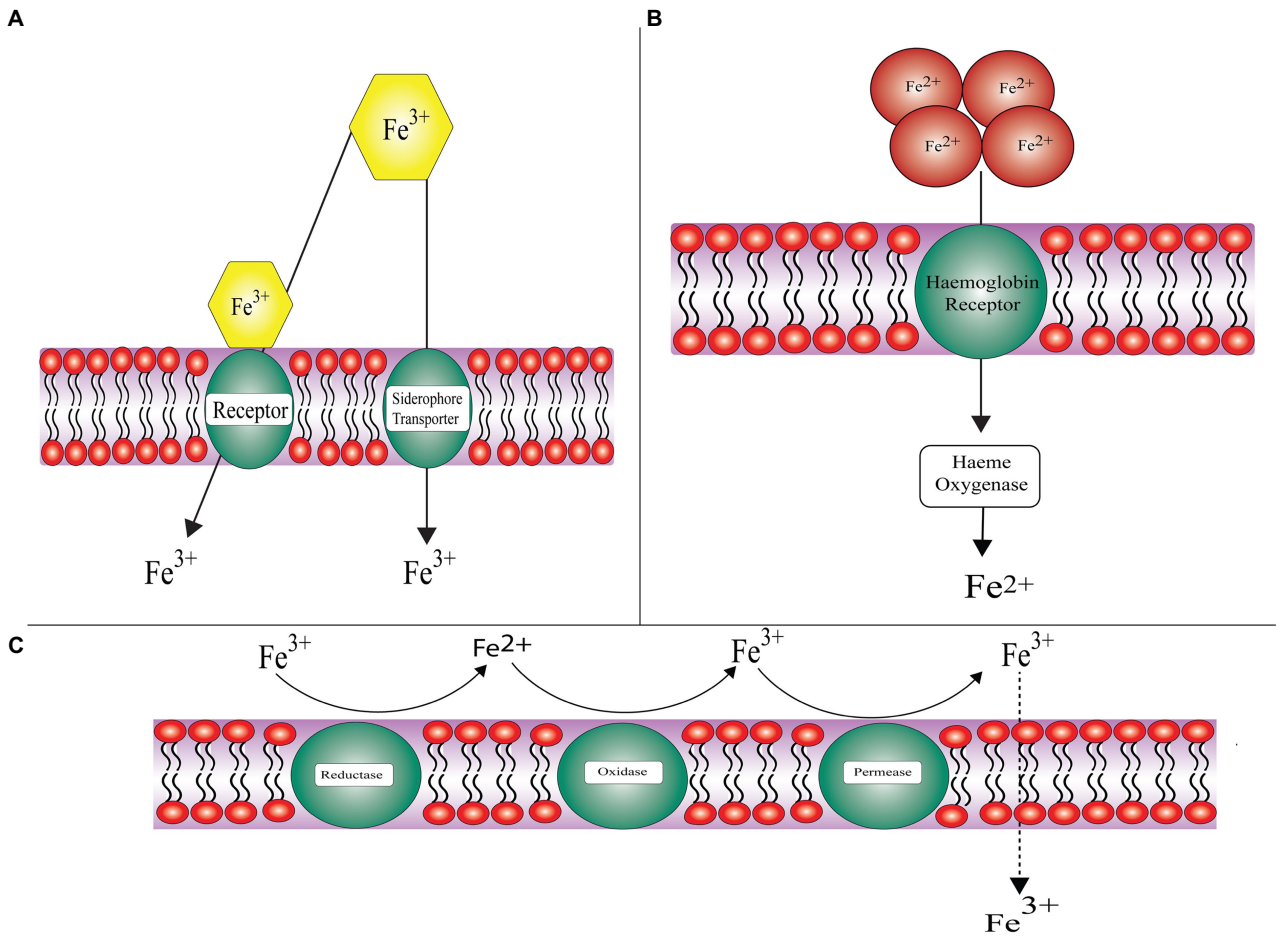
General approaches employed by pathogenic fungi for acquiring iron **(A)** Siderophore uptake system: acquisition of iron from siderophores and xenosiderophores, **(B)** Hemoglobin utilization: chelation of iron from heme proteins and hemoglobin, and **(C)** Reductive system: subject to assimilating iron through redox reaction and further transportation into cytoplasm dictated by iron permeases.

Iron is an essential micronutrient required for the progression of infection as it facilitates cell proliferation and development. Free iron is typically sequestered by transferrin, ferritin, and lactoferrin (Howard, 1999). A key virulence determinant possessed by Mucorales includes the capability to take up iron from the host cell. In order to accomplish this, the pathogen adopts various strategies, which include utilizing siderophore, heme, and free iron acquisition systems (Challa, 2019). The mechanism of iron acquisition by pathogenic fungi is shown in Figure 2.

#### Reductive System for Iron Uptake

Fungal pathogens like Mucorales, including *Rhizopus arrhizus*, *Lichtheimia corymbifera*, and *Mucor circinelloides* have a high affinity iron uptake system. *FRE1* and *FRE2* encoded ferric reductase membrane bound enzyme facilitates the reduction of iron, i.e., ferric (Fe^3+^) to ferrous (Fe^2+^). *FET3* encoded multicopper ferroxidase directs the re-oxidation of the insoluble ferric ion. *FTR1* encoded iron permease imports the insoluble ferric ion (Bairwa et al., 2017).

#### Hemoglobin Utilization

About 60–70% of iron binds to the heme moiety in hemoglobin of host. Some other proteins of the host that bind iron include lactoferrin, haptoglobin, hemopexin, lipocalin-1, and lipocalin-2. These proteins reduce the availability of iron to invading pathogens. An intracellular protein ferritin binds to iron and is the second largest reservoir of iron. It is composed of two subunits: L-ferritin (Ftl) and H-ferritin (Fth; Hare, 2017). Generation of toxic components and accessibility of iron to pathogen is forestalled by sequestering iron to explicit proteins (Stanford and Voigt, 2020). The two *R. oryzae* homologs of heme oxygenase were revealed by the *Rhizopus* genome project. These help the pathogen in uptake of iron from hemoglobin of the host. Intracellular heme uptake is promoted by *FTR1* in *R. oryzae*, which serves as an intracellular membrane permease further proceeded by degradation of heme to release ferric ions intracellularly. *SreA*, a transcriptional regulator assists *R. oryzae* in acquiring iron from the host (Ibrahim et al., 2008).

#### Siderophore Uptake

The third mechanism by which iron is acquired by the pathogen from accessible sources in the host and environment is a non-reductive pathway. Siderophores are low molecular weight (typically less than 1kDa) small iron chelators produced by bacteria or fungi having high affinity for iron.

On the basis of chemical nature of group donating oxygen ligand for Fe^3+^, they are categorized into three classes namely: catecholates, hydroxamates, and alpha-hydroxy carboxylates (Miethke and Marahiel, 2007; Wilson et al., 2016). Rhizoferrin being an intrinsic siderophore secreted by *Rhizopus* (belonging to the polycarboxylate family) helps in iron acquisition *via* receptor mediated and energy dependent mechanism (Thieken and Winkelmann, 1992). *Rhizopus* needs intrinsic as well as extrinsic siderophores for iron uptake (Morales-Franco and Nava-Villalba, 2021). The project on *R. oryzae* genome sequencing has revealed the possibility of presence of 13 siderophore permeases acting as siderophore receptors. The exact mechanism by which Rhizoferrin takes up iron is still not known (Boelaert et al., 1993). *X*-ray crystallography studies revealed the structure of rhizoferrin – around the chiral center two citric acid residues having R,R configuration are attached with the aminobutane backbone (Ibrahim et al., 2008). Rhizoferrin is unable to fulfill the iron requirement of the pathogen as it is incompetent in acquiring iron from serum because the gene responsible for producing the enzyme non-ribosomal peptide synthase that secrets common siderophore is absent (Boelaert et al., 1993). Deferoxamine, a xeno-siderophore helps in the chelation of iron from the protein, i.e., transferrin results in the formation of an iron-deferoxamine complex known as ferroxamine. With the help of fungal receptors Fob1 and Fob 2, this complex binds to the fungal surface; as a result of which ferrous ion is liberated by reduction at the fungal cell surface (Liu et al., 2015). Further, copper oxidase oxidizes ferrous ion to ferric ion on the fungal surface and later on transports it *via* high affinity iron permease (*FTR1*; Boelaert et al., 1993; Ibrahim et al., 2010).

## Epidemiology

Rhino-Orbito-Cerebral Mucormycosis (ROCM) is one of the most prevalent forms of Mucormycosis in India (Prakash and Chakrabarti, 2019), followed by pulmonary and cutaneous forms (Patel et al., 2020); though in developed countries, pulmonary mucormycosis is more prevalent (de la Fuente et al., 2012). Cutaneous form of the disease majorly develops in patients with burns or trauma (Prakash and Chakrabarti, 2019; Patel et al., 2020). In India, detection of renal mucormycosis is a rather unique clinical phenomenon, especially in healthy individuals (Chakrabarti and Singh, 2014). Chakrabarti and coworkers showed an expanding pattern in number of cases from a solitary community at continuous time periods, during 1990–1999 annual occurrences was 12.9 cases per year which increased to 35.6 cases per year during 2000–2004; and reached 50 cases per year during 2006–2007. It rose from an annual incidence of 25 cases every year in 1990–2007 to 89 cases per year in 2013–2014 (Prakash and Chakrabarti, 2019). A study was conducted across 12 centers in India wherein major aspects related to mucormycosis like predisposing factors; management, microbiology, and geographic profile were observed. About 465 subjects were included in total, where majority of the cases reported were of ROCM type (67.7%), followed by pulmonary, cutaneous, and others. The main risk factors included diabetes mellitus (DM; 73.5%), followed by hematological malignancies (9.0%), transplant (7.7%), and others. Diabetes mellitus was the major factor favoring the infection of all forms of mucormycosis. Several single center studies have suggested that mucormycosis epidemiology in India is different from the developed countries (Prakash and Chakrabarti, 2021). According to a study conducted at 12 tertiary centers in India from 2016 to 2017, uncontrolled diabetes mellitus is the most common risk factor for mucormycosis, excluding cutaneous and renal mucormycosis. A study suggested that the data recorded, showed a mortality rate of 52.0% in 90 days (242/465 subjects; Patel et al., 2020).

## Types of Mucormycosis

On the basis of anatomical site involved in the infection, mucormycosis is categorized into the following types: (a) ROCM, (b) Pulmonary mucormycosis, (c) Disseminated mucormycosis, (d) Cutaneous mucormycosis, (e) Renal mucormycosis, and (f) Gastrointestinal mucormycosis other types categorized as (g) Miscellaneous including infection of ear, parotid, urinary bladder, bones, heart, and lymph nodes (Prakash et al., 2019). By and large, people suffering from some underlying diseases are infected with ROCM and pulmonary mucormycosis, on the other hand cutaneous mucormycosis is more prevalent in immunocompetent patients. Disseminated mucormycosis is the spread of disease *via* the bloodstream and commonly affects the lungs and brain.

### Rhino-Orbito-Cerebral Mucormycosis

Rhino-orbito-cerebral mucormycosis is used to define Mucorales infection of the head and neck region. It is usually detected in immune-compromised patients. In the early stages, it infects the palate (root of the mouth) or paranasal sinuses; then spreads to the orbital region and subsequently reaches the brain if not diagnosed timely. Pain in the eye, ophthalmoplegia, poor vision, orbital cellulitis, necrosis, and ptosis etc. are some of the ophthalmic signs and symptoms associated with ROCM. The common non-ophthalmic manifestations are mucosal necrosis (nasal and oral); facial swelling, sinusitis, leukocytosis, facial numbness, mental status change, facial necrosis, and fever (Vaughan et al., 2018). Dark necrotic region is the hallmark sign of mucormycosis. ROCM is more commonly seen in people suffering from diabetic ketoacidosis (DKA) or uncontrolled diabetes mellitus. Individuals with underlying malignancies, other risk factors, those undergone solid organ transplants (SOT) or hematopoietic stem cell transplant (HSCT) are also vulnerable to this disease (Petrikkos et al., 2012).

Computed Tomography (CT scan) and MRI may help determine the extent of infection by studying bone tissue erosion, sinusitis with lesions, mucosal thickening, and damage to bones in maxilla, nasal septa, mandible, and orbit during paranasal mucormycosis (Petrikkos et al., 2012). High risk patients ought to undergo biopsy analysis of suspected regions of infection. Unresponsiveness to broad spectrum antibiotics during high fever is generally observed in patients infected with ROCM. Diagnosis of ROCM usually requires histopathological evidence. A meta-analysis of cases of this fungal infection revealed that *Rhizopus* species is most commonly associated with ROCM form (Prakash et al., 2019).

### Pulmonary Mucormycosis

After ROCM, lungs are the next most common sites of invasion by the pathogen, causing pulmonary mucormycosis. In a few cases, hemoptysis, pleuritic chest pain, and dyspnea were additionally noticed and non-productive cough was found to be a common symptom. Immunocompromised patients accounted for maximum number of cases, which may be a result of hematological disorders or organ transplant. In a significant number of cases, diabetes mellitus has been detected as underlying disease (Tedder et al., 1994; Lee et al., 1999). Infiltration of the lungs (58–96%), presence of multiple nodules, cavities (6–37%), pleural effusion (6–21%), lymphadenopathy (3.3%), and pneumothorax (1–3%) were some of the observations in imaging. Only 9.8% cases showed the reverse halo sign, which is generally a characteristic sign of mucormycosis. Patients with heme malignancy and suffering from pulmonary mucormycosis underwent CT scan imaging, where Chamilos et al. (2005) noted that multiple lung nodules and pleural effusion on initial CT scans were independent predictors of pulmonary mucormycosis. Post pulmonary tuberculosis has been reported as a prominent risk factor associated with pulmonary mucormycosis (Prakash and Chakrabarti, 2019). *Cunninghamella* sp. is generally found associated with pulmonary or disseminated type of disease.

### Cutaneous Mucormycosis

Cutaneous mucormycosis develops in immunocompetent hosts (43–67%) infected due to trauma or breach of skin (Skiada et al., 2011). Based on the extent of infection, cutaneous mucormycosis can be categorized as localized if it affects only the skin or deep extension if it involves muscle, bones, or tendons (de Oliveira-Neto et al., 2006). Cutaneous mucormycosis can spread gradually or it may lead to gangrene and dissemination due to raging spread (Hampson et al., 2005). Necrotic eschar encompassed by erythema is a typical manifestation of cutaneous mucormycosis and an insignificantly small erythematous macule could get disseminated in immunocompromised patients (Hocker et al., 2010). Significant concerns in cutaneous mucormycosis include penetrating trauma, and some minor concerns involve intra-muscular injection, surgery, open wound trauma, accidents, contaminated dressings, etc. (Roden et al., 2005; Suryanarayan Rao et al., 2006; Simbli et al., 2008; Skiada et al., 2011; Jeong et al., 2019). Visual diagnosis may include appearance of nodules, blisters, necrotic ulcers, pustules, etc.(Kobayashi et al., 2001). Occurrence of cutaneous mucormycosis in children is around 27%, which is around 19% of all cases (Roden et al., 2005; Petrikkos et al., 2012). A meta-analysis confirmed that *Saksenaea* sp. and *Apophysomyces* sp. are commonly associated with cutaneous mucormycosis (Prakash and Chakrabarti, 2019).

### Gastrointestinal Mucormycosis

Gastrointestinal mucormycosis is Mucorales infection of any part of the gut. It is seldom diagnosed in living patients because of its highly non-specific presentation, which requires major suspicion leading to endoscopic biopsy. Its mortality rate is extremely high, approximately 85% (Roden et al., 2005). Infection is caused by ingesting pathogenic organism *via* food items like fermented milk and/or bread products (Rogers, 2008). Majority of risk factors include SOT (52%), neutropenia (38%), broad spectrum antibiotics (37.1%), hematological malignancies (35%), and diabetes mellitus (12.2%). In adults, DM and peritoneal dialysis contribute to majority of risk, whereas malnutrition and use of broad-spectrum antibiotics are of particular concern among children. The most well-known site of infection includes bowel (64.2%). The patients generally report symptoms like stomach pain, diarrhea, abdominal distension, and gastrointestinal bleed (Dioverti et al., 2015; Kaur, 2018; Prakash and Chakrabarti, 2019). The infection may be associated with bleeding of upper GI tract (Echo et al., 2005; Geramizadeh et al., 2007). Patients with low levels of neutrophils may also show symptoms of neutropenic fever, typhlitis, and hematochezia. GI infection by Mucorales can also spread to pancreas, spleen, and liver. It can further enter bowel walls and blood vessels, leading to GI hemorrhage, bowel perforation etc., thus causing death (Suhaildeen et al., 2017). Neonatal GI infection can manifest as enterocolitis and is often associated with late or poor diagnosis and high case fatality (around 78%; Francis et al., 2018).

### Disseminated Mucormycosis

Mucorales are capable of invading blood vessels and can consequently enter hematogenous paths. Dissemination occurs most commonly in lungs; this is followed by CNS, sinus, liver etc.(Skiada et al., 2011). Recipients of SOT and individuals with hematological malignancy are more prone to disseminated mucormycosis (Jeong et al., 2019). Individuals with neutropenia, iron overload, or profound immunosuppression, leukemia, and the ones receiving deferoxamine are prone to disseminated mucormycosis. Fatal cases of disseminated mucormycosis are related with the usage of self-monitoring blood glucose equipment, which showcases a subtle presentation of disseminated mucormycosis. Tissue cultures of immunocompromised patients gives no clear skin findings, which is a major drawback in case of dissemination of the disease (Hocker et al., 2010).

### Miscellaneous Mucormycosis

Isolated renal mucormycosis affects intravenous drug users. Patients undergoing renal transplant in warmer climates also may suffer from renal mucormycosis (Stas et al., 1996; Weng et al., 1998; Chkhotua et al., 2001). Osteomyelitis of femur, tibia, humerus, and sternum has been reported and is commonly noted after surgical intervention (Wanishsawad et al., 1996). Hematogenous osteomyelitis is extremely rare (Holtom et al., 2000).

## Predisposing Factors

There are numerous predominant underlying conditions conducive in making a host more vulnerable to fungal agents suspected to cause mucormycosis. The factors like ketoacidosis, uncontrolled diabetes, excessive use of corticosteroids and immunosuppressant drugs, increased number of transplantation surgeries, direct epidermis exposure to the spores of causative agents that happens either due to trauma or burns, hematologic malignancies, and deferoxamine therapy are majorly responsible for mucormycosis (Spellberg et al., 2005).

Recently, SARS-CoV-2 mutants have been implicated in aggravating the disease spread. It is increasingly becoming a cause of concern due to prolonged stay of patients in hospital wards and the sensitive healthcare systems and poor hospital management makes the immunocompromised host more vulnerable. In a crucial study by Skiada et al. (2020), it has been observed that distinctive topographical regions contribute to different factors attributed to outbreak of infection. For instance, malignancies were associated with a majority of cases reported from European countries and in Middle East and Asian countries the major predisposing factor was diabetes. It was additionally highlighted that a specific manifestation is due to some risk factor targeting a particular tissue, like cutaneous mucormycosis is the result of trauma or burns, diabetes majorly causes ROCM, while neutropenia and malignancy lead to pulmonary mucormycosis (Skiada et al., 2020).

Owing to the changing lifestyle patterns, the cases of DM are dramatically increasing in developing countries. It is reported that diabetes is perhaps the most significant risk factor for the prevalence of MCR with 40% of the total cases with 20% of cases reported from ketoacidosis patients, which becomes the second highest factor followed by malignancies and transplantations (Jeong et al., 2019).

### Use of Steroids and Immunosuppressants

A wide range of medicinal drugs is available to combat, control, and treat various deadly diseases nowadays. Major classes of these medicines include steroids, corticosteroids, and immunosuppressant. Steroids are the inbuilt hormones produced naturally by the body’s ductless glands and behave as chemical messengers enabling regulation of the endocrine system. Corticosteroids structurally resemble the cortisol hormone, which is a steroid hormone synthesized by the adrenal glands and aids in the normal functioning of the immune system and metabolism. Besides, it also helps in stress regulation. Intake of corticosteroids by artificial means suppresses the immune response of the body by inhibition of the inflammatory pathways, further leading to higher susceptibility to various fungal pathogens (Skiada et al., 2018).

It has been observed in certain retrospective studies that invasive pulmonary mucormycosis was caused by short periodic courses of corticosteroid intake by patients who were previously suffering from mild diabetes which was kept under control. Indeed, even 15 days of short-dose course can result in some serious consequences like phagosomal fusion of the macrophages present in the bronchoalveolar region (Hoang et al., 2020). The steroids widely used to treat autoimmune dysfunctions, malignancies, and for post-transplantation surgeries can lead to phagolysosome formation, poor migration, and destruction of macrophages (Kontoyiannis and Lewis, 2011). Multiple high dose courses can lead to fatal fungal infections that can only be treated by aggressive surgeries to remove the affected organs.

It is believed that populace with higher predominance of diabetes and undergoing immunosuppressant courses are more prone to such invasive mucormycosis infections with greater mortality rates. Steroids drop our immunity, which further triggers a rise in cases of this fungal infection (Hoang et al., 2020). Due to increased intake of steroids to treat severe and critically ill patients suffering from the novel COVID-19 disease peaking these days, black fungus cases have been sharply rising. Therefore, it is advisable not to misuse steroids or any other drug and to only take the prescribed doses in order to prevent the spread of invasive infections.

### Post Transplantation Surgeries

In various prospective and retrospective studies carried out by researchers, it is observed that recipients of the HSCT and SOT surgeries succumb to various invasive fungal infections (IFIs). These patients behave as immunocompromised patients and become potential hosts for Mucorales (Lanternier et al., 2012).

The risk further increases in patients with diabetes, renal failure; these patents are prescribed high dosages of antifungal drugs voriconazole or caspofungin, that fall into the category of old azoles used to treat fungal infections (Singh and Sun, 2008). Studies suggest that the global risk factor of infection in the SOT recipients is about 14% (Jeong et al., 2019). For the patients who have received liver organ transplantation surgeries, high iron concentrations make the recipient susceptible to such opportunistic invasive fungal infections, since iron is responsible for growth of mucorales species (Singh and Sun, 2008).

The analytical evidences of disease estimation in patients undergoing HSCT surgeries are around 12% in countries like France. On the other hand, the incidences reported from developing countries such as India, Iran, and the South American nations are as low as 1–2% for the HSCT patients. For the years in between 2001 and 2006, the data for IFI epidemiology caused by Mucorales in a transplanted host were analyzed in a progressive surveillance record done by the Transplant-Associated Infection Surveillance Network (TRANSNET), who considered MCR incidence cases from across 23 US centers (Park et al., 2011).

### Diabetes and Hyperglycemia

The primary factor responsible for black fungus infection is DM. Data reveals that in countries like India, Iran, and Mexico, the percentage of the people suffering from diabetes and susceptible to MCR infections is around 75%; whereas this is 52% for the US population (Prakash and Chakrabarti, 2019). Small European countries like Italy, France, and Lebanon have reported 20–23% of DM related IFIs as shown in a study by European Confederation of Medical Mycology (ECMM; Vaezi et al., 2016).

In case study of a patient suffering from diabetes, MCR of faciomaxillary region with fungal infection in the nose and sinus portion was observed. Palatal ulceration is widely seen in such cases, which further leads to necrosis. The host being immunocompromised due to the preexisting disease becomes less resistant to the invasive fungal infection and becomes a potential host (Afroze et al., 2017).

Uncontrolled type 2 diabetes mellitus weakens our immunity; subsequently patient becomes a suitable host for many infections. Fungal proliferation is stimulated by increased blood sugar levels. Chemotaxis and phagocytic efficiency is also depressed and therefore, mucorales survive the acid rich environment due to failure of host’s natural response of fungal killing. Besides sinuses, the rhino-orbito-cerebral region is greatly affected in diabetes-related mucormycosis; thereafter, the pulmonary and cutaneous regions are affected. In the patients suffering from DM, the signs for aggressive invasive fungal infections include facial symptom like pain and purulent nasal discharge (Rammaert et al., 2012), whose delayed diagnosis can lead to further complications making it difficult to treat with antifungal therapies. In such a situation, affected region would need to be surgically removed.

Black fungus can be rightly called one of the unmasked diseases of diabetes (Patel et al., 2020). Increased level of glucose could act as good nutritional food source for development and growth of the fungal species. Garret’s succession theory emphasized that fungal sugar decomposition is done by sugar fungi as the primary pathogens that are followed by cellulolytic and ligninolytic fungi and eventually leads to growth of secondary pathogens responsible for the invasive infections (Pandiar et al., 2021).

Hyperglycemia is the cause of concern in modern day lifestyles. The global lockdown contributed in upsurge of hyperglycemia cases, since work from home has led to lack of physical activities. It is also estimated that the intake of immunosuppressive drugs enhances the blood sugar levels, causing type 2 diabetes mellitus.

### Ketoacidosis

Many patients suffering from DM also suffers from DKA. Indigenous geographical diet patterns and demographic features of a region affect the prevalence of this disease. For instance, in India, 90% of DKA cases are reported from the northern regions and the remaining 10% from the southern regions of the country (Prakash and Chakrabarti, 2019). DKA disrupts phagocyte functioning, halts intracellular killing by suppression of various non-oxidative and oxidative mechanisms, and also disrupts chemotaxis. The patients suffering from DKA display elevated serum iron concentration released from transferrin, ferritin, and lactoferrin binding proteins in acidic conditions. The Mucorales uptake iron with the help of iron permease due to high affinity for copper (*rFTR1*) and copper oxidase enzyme (Sarvestani et al., 2013).

*Rhizopus oryzae* is responsible for causing mucormycosis in keto-acidosis patients, since these pathogens produce the keto-reductase enzyme, enabling them to utilize ketone bodies from the host. The growth of hyphae from *R. oryzae* is attributed to the disrupted host defense mechanism, which is caused by the alteration of transferrin binding to iron (Artis et al., 1982). Dysfunctional phagocytic behavior in hyperglycemic DKA, disrupted host-pathogen defense mechanism, increased iron concentration in serum leading to transferrin binding defects, and the ketone reductase activity in hyperglycemic ketoacidosis leads to MCR (Thomas et al., 2020). All these factors are cumulatively responsible as the key causes of incidence of mucor cases in the patients suffering from ketoacidosis.

### Malignancies

Hematological malignancies alone are responsible for major cancer deaths. Patients with mucormycosis and HM have predisposition to developing acute lymphoblastic leukemia, non-Hodgkin’s Lymphoma, acute myeloid leukemia, myelodysplastic syndrome, and various other rare types of malignancies. The patients affected by neutropenia are at a greater risk of contracting IFIs (Skiada et al., 2020). According to the global data provided by the WHO, the number of fatalities caused by malignancies is about 10 million. Leukemia accounts for about 474,519 (2.5%) of global cancer cases whereas multiple myeloma, lymphoma cases are 176,404 (0.9%) and 83,087 (0.4%), respectively (Sung et al., 2021).

In a study, Kara et al. (2009) observed that some patients who suffered malignancies like lymphoma, acute leukemia, and developed neutropenia and become prone to mucormycosis. Research has proven that the most frequently affected sites are the sinuses in 95% of cases and lungs in remaining 5% of reported incidents. The neutrophil count of those patients went below the normal levels. The reasons associated with the spread of infection are profound neutropenia for prolonged duration after myeloablative treatments for hematologic malignancies. About 90% of patients with acute leukemia reported mucormycosis along with a comparatively higher mortality rate of 55% than other malignancies (Kara et al., 2009).

Hematological malignancy patients not only act as potential hosts to fungal agents but also suppress the immunity of the body against many other infections and lead to serious cardiac toxicity. Therefore, it is advisable to undergo adjunctive treatment therapies other than chemotherapy. One of the possible solutions is to find a suitable and timely donor to help recover the neutrophils lost. Antimicrobial prophylaxis against these fungal agents can prevent the spread of infection.

### Increased Zinc and Iron

Metal ions have a profound effect in triggering a particular disease. The role of zinc and iron has been associated with the spread of mucor related infections. The increased iron levels make an individual more vulnerable to infection. The reason attributed to this is the iron overload and the defective host-pathogen defense mechanism (Artis et al., 1982).

Previous studies have reported increased incidences of invasive mucormycosis among patients who received deferoxamine iron chelators, which behave as siderophores and provide iron to the fungal species. It is originally bound to the transferrin and ferritin and is unavailable to pathogen. Increased iron uptake further leads to elevated iron serum levels (Spellberg et al., 2005). Availability of iron increases when host is suffering either from ketoacidosis or has undergone transplantation surgeries. It is also proven that the metal acts as an important source of nutrition for the microbes. The medical factors related to iron overload contribute to invasive fungal infections. The host response to the microbes ceases and the virulence of causative agent also enhance due to increased iron concentration (Singh et al., 2009).

Other than its role in pathogenesis of the infection, iron also assumes a critical role in immune system of the host. Both adaptive and innate responses consist of iron as an intermediate component. Interferon mediated pathways of cell lines are inhibited by overload of iron. The phagocytic activity of the neutrophils declines in the presence of high iron concentrations. The infection progressed by changes in the CD4 cell responses, eventually leads to increased incidences of the disease (Singh et al., 2009).

Recent studies have shown that in current situations, the spread of mucormycosis is also due to the increased zinc concentrations in the drugs being used to treat COVID. However, the exact role and mechanism of the proposed hypothesis are under investigation. The use of zinc chelators is the probable cause as it is a well known fact that zinc starvation is related to inhibition of Mucorales species (Leonardelli et al., 2019). Due to prevalence of SARS-CoV-2 mutants across the globe, these medications are used widely. It can be taken into account that to forestall the upsurge of invasive fungal cases, zinc depletion therapy could be a way forward to combat and control the infection.

### COVID-19 Associated Mucormycosis

A total of 20,908 cases of mucormycosis with 1,376 confirmed deaths have been reported from India so far as July 31, 2021 as per the reports of Ministry of Health and Family Welfare. The active cases were more than 28,000 and were declared as “black fungus epidemic” (Singh et al., 2021). Around 80% of the cases had the history of diabetes, around 14.9% were suffering from DKA, and about 86% of the patients were exposed to corticosteroid treatment for COVID-19 disease. Around 78.9% of the cases were reported in males, indicating the predominance of the infection. COVID-19 Associated Mucormycosis (CAM) affects the sinuses in 88.9% of the cases, followed by the rhino-orbital cerebral region in 56.7% of the reported cases and the rate of mortality was 30.7% (Singh et al., 2021). In a recent study, with a COVID patient, the CT image of the face displayed the mucosal thickening in the Sino-maxillary and ethmoid sinus part along with other complications related to opacification (Werthman-Ehrenreich, 2021).

COVID-19 Aassociated Mmucormycosis is attributed to aseptic and thermo-tolerant properties of the pathogens responsible for causing the infection, as they can even endure relatively high temperatures. Other factors which link spread of the fungal disease to COVID patients or survivors include endothelial damage, increased ferritin levels, multiplied iron and zinc levels, and elevated hyperglycemia. The clinical symptoms of the disease include obstruction in nasal region, periorbital, or the buccal swelling alongside the emergence of black spots at the affected part. SARS-CoV-2 activates a cascade of biochemical responses in the patient’s body (Kaur et al., 2021; Periwal et al., 2021) that further acts as a desirable factor for certain opportunistic infections, for instance, mucormycosis (Pandiar et al., 2021). The research done so far suggests a strong link between COVID-19 and invasive fungal disease by means of “endothelialitis” i.e., the endothelial lining impairments.

A hypothesis proposed by the Bellanger et al. (2021) states that steroids used for treating SARS-CoV-2 are conducive to mold development in the suspected host, which further grows to secondary fungal infections, ultimately leading to loss of vision in critically ill COVID patients along with hearing impairments and eventually becoming the reason of death. Hence, it is a must to look into the cause and suggested treatment of the MCR, so that it does not complicate the risk of COVID related co-morbidities. There is also a need to look for novel strategies to get rid of the infection.

### Poor Healthcare

A large number of MCR cases have been reported with prime cause of invasion being poor healthcare systems. The healthcare-associated mucormycosis can be attributed to use of certain non-sterilized medical tools and sharing common wards for housing patients with different diseases that can spread infection. It is also observed that the technologies in use for diagnostic purposes are shared between many visitors, which can be a possible spreader of the invasive infections. High incidences of MCR infection are reported for patients with prolonged stay at hospitals due to health issues (Hartnett et al., 2019). The infection by this opportunistic pathogen is also related to exposure of the person to non-sterile healthcare equipment like uncleaned bandages, patches of nitroglycerin, and adhesives (Petrikkos et al., 2003). Additionally, it was highlighted that the use of wooden tongue depressors and the ostomy bags can also complicate the mucor outbreak. It was reported that the consumption of packaged food prepared long time ago, supplements uptake and allopurinol tablets can further add to the increased number of cases (Cheng et al., 2009). Linens is commonly used in practice gets contaminated while in storage and transportation, hence contributing to mucormycosis related to healthcare (Duffy et al., 2014). Another factor contributing to the infection is utilization of certain procedures and medical equipment such as the catheters; tube insertion. Eventually, the breaks in skin induced by the use of finger sticks and infusion pumps help the mold to grow even if the probability of occurrence is near negligible (Hampson et al., 2005). Dental and transplantation surgeries also lead to complications of infection by making the recipients vulnerable to molds. Other factors are related to the contaminated environmental conditions of the hospitals either due to construction works or can also be due to the water leakage or the negative or positive pressure rooms or spore aerosolization (Hartnett et al., 2019).

### Other Underlying Factors

Many other underlying factors are thought to favor the spread of the disease. Some researchers have associated antifungal prophylaxis (Lamoth et al., 2016). Many others have linked this infection to chronic alcohol intake, autoimmune diseases, renal failures, lower body weight in infants, liver dysfunctions, intravenous drug use, malnutrition, and AIDS (Jeong et al., 2019). Nucci and coworkers suggested that 6.9% of the MCR patients had post-pulmonary tuberculosis whereas 8.9% of them had chronic kidney diseases (CKD; Nucci et al., 2019). Chronic Obstructive Pulmonary Disorder (COPD) is an underlying factor reported among 7–46% patients (Patel et al., 2020). Steroid therapy was also seen as a factor responsible for MCR infection. Some studies also attributed the prevalence of MCR infections to overuse of voriconazole, which is commonly prescribed for the treatment of invasive fungal infections. Nonetheless, it must be considered that the spectrum of this invasion is dependent on specific mold and its epidemiology. Also, the characteristics of patients do have a significant role in deciding the incidence of fungal growth and the outbreak (Lionakis et al., 2018).

### Disease Prevention

A protective environment plays a very important role for prevention of incidence of this disease. According to the guidelines published by CDC, at individual levels, one should avoid places involving construction activities and if unavoidable, it is preferred to wear N95 respirators or masks. Direct contact with soil and dirt must be avoided; one should wear full sleeves, gloves shoes, and long pants to avoid close contact. It is a must to regularly clean and sanitize the exposed skin. If a patient has undergone any sort of surgery, proper antifungal medications must be taken after prescription from the doctor (Brizendine et al., 2011; Rogers et al., 2011; Davies et al., 2017). It is necessary to maintain good personal hygiene, take regular baths, and avoid overcrowded regions.

The guidelines also recommends that during stay at hospital, the patient undergoing any treatment or surgery needs to be in a well isolated and protective environment having well equipped HEPA filters and positive pressure, which is less than or equal to 2.5 Pa relative to that of the corridors. In order to avoid any kind of mold growth, periodic health assessments, and investigations needs to be done with full health record maintenance of patients. The molecular diagnostic tools and other areas must be sterilized from time to time (Hartnett et al., 2019).

The disease can be prevented if detected in early phase of its infection and the underlying factors should be removed. Clinical manifestations and pathogenic mechanisms of various underlying disease conditions are described in Table 1. The controlled levels of blood glucose can help prevent both diabetes and MCR as the pH levels can be maintained to normal levels and mold growth would be suppressed. The use of corticosteroid drugs and immunosuppressants must be immediately stopped as this is one of major contributing factor for mucormycosis. COVID care centers must be regularly sanitized and proper social distancing must be followed. Self-examinations for the disease are also advised and if any such manifestation appears, one must contact the doctors immediately.

**Table 1:** Clinical manifestations and pathogenic mechanisms of underlying conditions.

S.No.	Underlying factors	Manifestation in order of frequency	Pathogenic mechanism
1.	Diabetes/ketoacidosis	Rhino-orbital-cerebral > Pulmonary > Gastrointestinal > Cutaneous	Neutrophil activation and iron uptake for growth in acidic medium
2.	Malignancies	Disseminated; Pulmonary	Leukemia and prolonged neutropenia
3.	HSCT	Pulmonary >Rhino orbital cerebral>Gastrointestinal	Prolonged neutropenia
4.	SOT	Pulmonary > Cutaneous > ROCM > Disseminated > Gastrointestinal	Immune suppression and induced diabetes
5.	Iron overload	Disseminated > Pulmonary > ROCM > Cutaneous > Gastrointestinal	Uptake of iron for growth purposes
6.	COVID-19	Pulmonary > Cutaneous > Disseminated	Immune suppression, prolonged hospital stay
7.	Trauma/Burns	Cutaneous>Pulmonary>ROCM>GI > Disseminated	Direct cutaneous inoculation of spores
8.	Corticosteroids	Disseminated > Pulmonary > ROCM > Cutaneous > Gastrointestinal	Induced diabetes; macrophages and neutrophil damage
9.	Hospital acquisition	Cutaneous > Pulmonary > Disseminated > ROCM	Spore inoculation: prolonged hospital stay: unsterilized tools sharing direct exposure to spores of molds due to shared tools leading to necrotic lesions underneath the skin.

Adapted from Petrikkos et al. (2012).

## Signs and Symptoms

### Rhino-Orbital-Cerebral Mucormycosis

Sinusitis and periorbital cellulitis are one of the initial symptoms of mucormycosis (Dhiwakar et al., 2003). Other symptoms that can become extremely severe include swelling on one side of the face and numbness, nasal congestion, and palate or nasal bridge with black lesions. At times, the dissemination reaches the eyes and lead to fluid build-up, causing swelling (periorbital oedema), bulging or displacement of the eye (proptosis), and loss of eyesight and may ultimately causing paralysis or weakness in eye muscles (Hosseini and Borghei, 2005; Fouad et al., 2021).

### Pulmonary Mucormycosis

It is majorly caused by the inhalation of mucor spores and the infected parts include bronchi, alveoli etc. Symptoms consist of fever, shortness of breath, cough, and chest pain. In some cases, spitting or coughing of blood (hemoptysis) can also be a significant symptom of Mucorales infection (Lewis and Kontoyiannis, 2013; Farmakiotis and Kontoyiannis, 2016).

### Cutaneous Mucormycosis

It can be gradual or severe and sudden (fulminant). It is maximally seen as blisters or ulcers (necrosis) with blackened infected areas. It may also be seen as pain, heat, redness, or swelling of the area surrounding the wound. It is externally developed as a painful and hardened area of the skin with swelling. Fever may also be observed in affected individuals (Castrejón-Pérez et al., 2017; Suthananthan et al., 2017).

### Gastrointestinal Mucormycosis

It is majorly seen in the form of abdominal pain, nausea, GI bleeding, and hematemesis. Perforation, as in lesions, can develop in the stomach and intestine. Inflammation of peritoneum (peritonitis), severe pain in bowels due to lack of flow of blood (bowel infarction), and hemorrhagic shock can also affect certain individuals (Spellberg, 2012; Kaur et al., 2018).

### Disseminated Mucormycosis

It is generally observed in highly immune compromised people with medical conditions, which makes it difficult to distinguish symptoms of fungal infection from symptoms due to some other ailment. In case of infection of the brain, symptoms may include changes in mental conditions or coma. It may spread to other organs like brain, kidney, spleen etc. Disseminated mucormycosis consists of various signs and symptoms depending greatly upon the organ involved. It can also disseminate to heart valves (endocarditis) or bones (osteomyelitis; Walsh et al., 2012).

## Transmission of Mucormycosis

**Figure 3:**
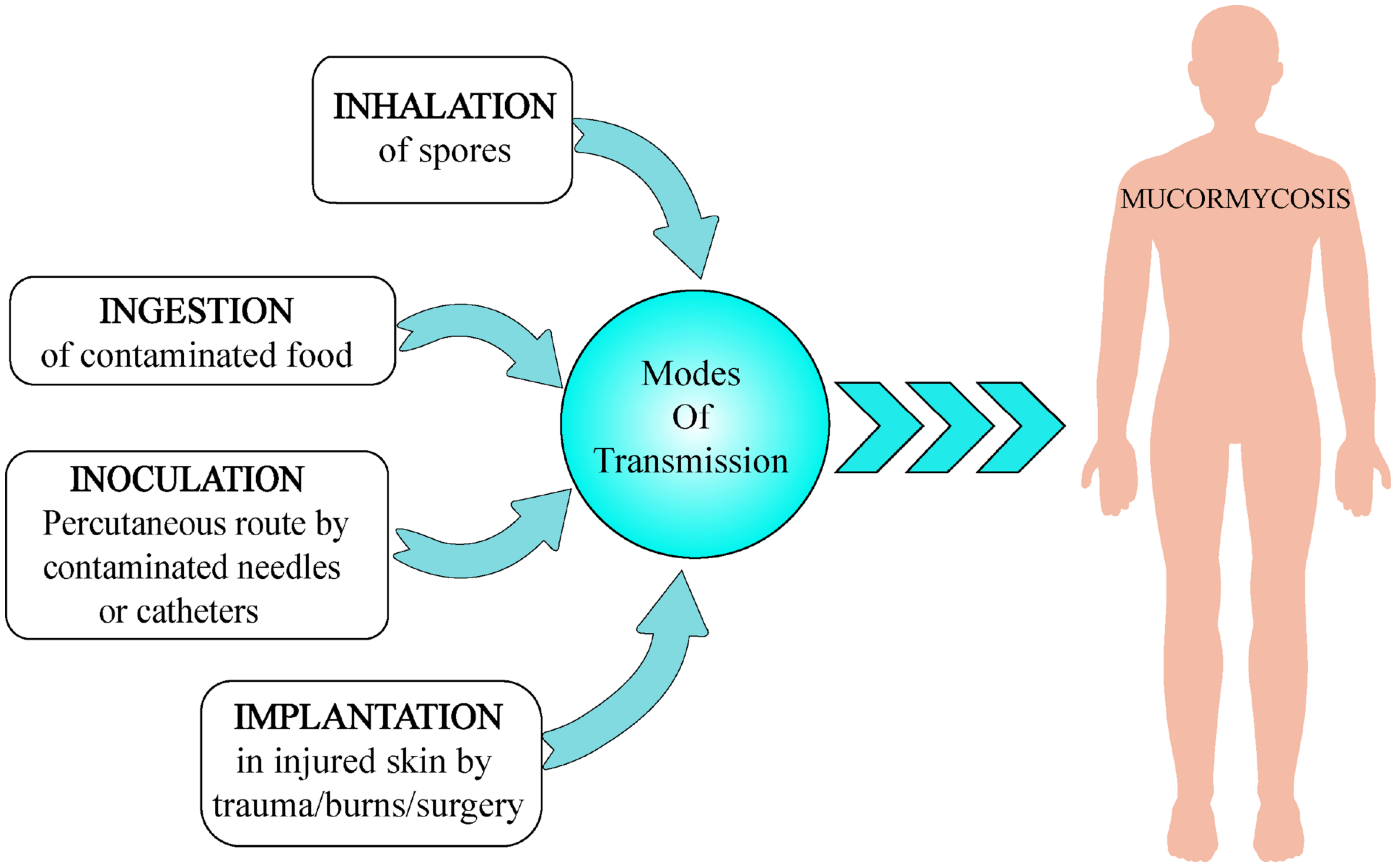
Various modes of transmission of mucormycosis include (a) inhalation, (b) ingestion, (c) inoculation, and (d) implantation.

Spores of Mucorales are omnipresent in nature and can be found in dead and decaying matter and soil. They cause no harm to immune competent patients in general, but an individual with weak compromised immune system gets severely infected on inhaling these mold spores (pulmonary, gastrointestinal, or sinus mucormycosis). It is a non-contagious disease. Sometimes, the spores can invade the body *via* incisions or open wound like a scrape or burn (cutaneous mucormycosis). It can also spread through ingestion of contaminated food (gastrointestinal mucormycosis) or through local inoculation (Binder et al., 2014). Various possible modes of transmission of mucormycosis are shown in Figure 3.

## Diagnostic Approaches

For good prognosis and disease resolution, early and accurate diagnosis of mucormycosis is important (Skiada et al., 2018) before angioinvasion occurs and necrosis becomes extensive, leading to dissemination (Ibrahim et al., 2007; Kontoyiannis and Lewis, 2011). It saves the patients from a disfiguring surgery in order to remove the necrosed tissue as curation and antifungal therapy alone is rarely successful (Millon et al., 2013). Presently, diagnosis of mucormycosis depends mainly on identification of morphological characteristics from cultural, histopathological, or radiological analysis (Bernal-Martínez et al., 2013). For species identification, characteristic structures such as type of sporangiospores or morphological features like columella shape, presence of an apophysis, stolon branching and organization, the presence of rhizoids and the shape, and size and structure of zygospores can be compared using fungal identification guides. Diagnosis for disseminated mucormycosis is extremely challenging, mainly because patients are ill from several underlying diseases. If there is any evidence of infarction, an in-depth examination should be done for cutaneous lesions to be biopsied and diagnosis of mucormycosis should be considered (Spellberg et al., 2005).

### Histological Analysis

Prominent infarcts, angioinvasion, and perineural invasion are the histopathological characteristics of mucormycosis. While using optical brightening agents in clinical specimens, it was observed that in contrast to *Aspergillus* hyphae, hyphae of Mucorales are not septate with flexible width having dimensions in the range of 6–25 μm. It was also visualized that they have an irregular ribbon like appearance and perpendicular branching/bifurcations. Some features of the order Mucorales include, abundant sporangiospores in a distinctly shaped columella inside a sporangium, non-septate mycelium, and telemorphic sporangiospores, often with ornamentation is a characteristic feature of (Hoffmann et al., 2013). These types of hyphae can be detected *via* biopsies of lesions. Biopsy may also include inflammation and polymorphonuclear infiltration often including eosinophils and plasma cells. Other indicators include necrosis with invasion of neutrophils, and presence of epithelioid cells highly suggestive of mucormycosis (Katragkou et al., 2014; Farmakiotis and Kontoyiannis, 2016).

### Computed Tomography and Magnetic Resonance Imaging

In case of ethmoid sinus infection, some imaging techniques like CT and MRI can detect the opacification, bone erosion, and obliteration of deep fascial planes (Skiada et al., 2018). Imaging studies are frequently negative initially or have only subtle findings. Commonly, CT scanning and MRI of the head or sinuses show slight thickening in sinus mucosa or thickening of the extra ocular muscles. Further, absence of deformities in sinus bones in spite of clinical sign of progressive disease is also common. To find an organized retro orbital mass is very uncommon in the diagnosis of ROCM (Talmi et al., 2002). High resolution CT scan is one of the best methods for determining the extent of pulmonary mucormycosis, even before the infection is seen in the chest X-ray (Spellberg et al., 2005).

### Culture Tests

Specimen cultures are widely used for identification at the genus and species level and also routinely used for antifungal susceptibility testing. Culturing along with CT guided percutaneous lung biopsies are considered essential for better diagnosis (Lass-Flörl et al., 2007). The observation that Mucorales grow on fungal culture media like potato dextrose agar (PDA) and Sabouraud agar incubated at temperature in the range of 25–30°C was made by Lackner and colleagues. Culture yield of *Rhizopus* sp. was improved in microaerophilic environment that resembles the conditions of infarcted tissue. Studies have shown that the conventional culture methods are not always reliable for this pathogen (Tedder et al., 1994). The fragile non-septate growth of fungi makes them vulnerable to physical damage during sample manipulation, which clearly explains the high number of negative culture results but positive microscopic results (Lackner et al., 2014).

### Molecular Analysis

Molecular approaches are employed for the identification at genus and species level and analysis of infection (Skiada et al., 2020). Primarily, the internal transcribed spacer (ITS) region is selected as the pan-fungal bar-code by the International Sub-commission on Fungal Barcoding and this forms the basis for molecular assays. Predominantly, PCR based approaches are preferred for proper analysis of the disease in its initial phases (Baldin et al., 2018). Molecular techniques are used as they are more rapid and reliable than the classical method for mycological identification (Dannaoui, 2009).

PCR based techniques include real-time PCR (qPCR) targeting 28S rDNA (Kasai et al., 2008), nested PCR combined with RFLP (Zaman et al., 2017), real time multiplex PCR targeting ITS1/ITS2 with specific probes for *Rhizopus* and *Mucor* species (Bernal-Martínez et al., 2013), and PCR/high-resolution melt analysis (HRMA; Alanio et al., 2015; Skiada et al., 2020). As compared to paraffin embedded tissue samples, these methods are well executed in fresh or deep-frozen samples. In the case of Formalin-Fixed Paraffin-Embedded (FFPE) samples, the analytical sensitivity is about 56–80% whereas in case of fresh tissue samples it is 97–100%.

For determining mucormycetes at species level, various loci like *FTR1* gene, ribosomal targets 18S, 28S, and ITS, and cytochrome b are being targeted in molecular assays. Molecular methods were earlier practiced on samples obtained from culture of biopsies or Broncho-alveolar lavage (BAL) and later on for fungal classification when cultures tested negative (Voigt et al., 1999; Schwarz et al., 2006; Millon et al., 2019). For identification of zygomycetes species, specific primers have been designed on the basis of variability between 28S and 18S DNA, 28S sequences being more conserved (Dannaoui, 2009).

Nowadays, non-invasive PCR based approaches are being followed for determining Mucorales DNA in samples (serum or plasma or urine) and prior differentiation of infection caused due to *Aspergillus* and Mucorales. Noninvasive procedures have more success rate than invasive procedures, as in case of invasive procedures, it is difficult to obtain samples from patients in ICUs and hematological malignancies (Millon et al., 2019).

### Molecular Diagnosis in Tissue Samples

Primarily, pan fungal PCR assays were used (Kidd et al., 2020) and this technique proved to be effective in successful detection of species in majority of cases (Voigt et al., 1999; Buitrago et al., 2013; Millon et al., 2019). Another approach involved the use of Mucorales specific primer tailored to unique species and genera (Bialek et al., 2005). This semi nested PCR targets the 28S sequence and is a steady option for the diagnosis of Zygomycetes. It also identifies the pathogen when the cultures test negative with a turnaround time < 48 h (Hammond et al., 2011). Further, this semi nested method is modified into high resolution melt curve analysis in real time PCR for the diagnosis of Mucorales (Hrncirova et al., 2010). This melt analysis preceded by real time PCR is considered a successful approach for detecting Mucorales (Millon et al., 2019). A conventional PCR assay established by Nagao et al. (2005) is based on partial ITS analysis for differentiating between species. This assay could not distinguish between *R*. *azygosporus* and *R*. *microsporus* and also it had low sensitivity (Nagao et al., 2005).

Hsiao et al. (2005) devised another method wherein probes were synthesized to differentiate between *R*. *arrhizus*, *L. corymbifera*, and *Rhizomucor pusillus*. This microarray technique based on oligonucleotides offers various advantages but it is cost ineffective and furthermore needs skilled personnel for data interpretation (Hsiao et al., 2005). In case of PCR – RFLP method, Nyilasi et al. (2008) developed a method wherein high affinity iron permease 1 (*FTR1*) genes were used as target and main aim was to differentiate among the clinically important pathogenic species of mucormycetes, which includes the *Rhizopus* sp. Although this method has high potential, huge cost and manpower expenditure make it a less opted molecular method (Nyilasi et al., 2008).

In a study by Hata et al. (2008) wherein they targeted cytochrome B genes in a semi nested RT-PCR followed by HMR analysis for further identification and discrimination of species mainly responsible for mucormycosis. Specificity and sensitivity can be enhanced by the combination of RT-PCR assays targeting 28S rRNA which helps in identification of the genus – *Rhizopus*, *Mucor*, and *Rhizomucor*, following HMR analysis (Hata et al., 2008). Millon et al. (2019) constructed a quantitative multiplex probe, focusing on clinically important fungi – *Mucor*/*Rhizopus*, *Lichtheimia sp*. and *Rhizomucor sp*. It mainly targets 18S rRNA and consists of three hydrolysis probes (Lackner et al., 2014). This assay has great potential for the detection of Mucorales as it is cost effective, specific and fast due to its turnaround time of about 3 h. This methodology is applicable for testing of Mucorales DNA in circulating blood/serum and also for screening patients with hematological malignancies (Millon et al., 2019). A different method for detection of a broad range of Mucorales species is the use of probe based (fluorescent labeled) real time qPCR. It is a sensitive technique and uses only one well of qPCR plate and for the identification of genera, sequencing is required (Millon et al., 2019). Proteins encoding spore coating *CotH* have potential to act as targets for facilitating diagnosis of infection caused by Mucorales. CotH genes are unique to fungi, which aid its penetration inside the host cell (Baldin et al., 2018). In a study by Baldin et al. (2018) they scrutinized whether PCR assay specific for CotH could identify Mucorales DNA of various species in different sample types. This test has given encouraging results in positive patients with specificity of 100% and sensitivity of 90% (Baldin et al., 2018; Kidd et al., 2020). The gene fragments of CotH could be obtained from various biological fluids like serum, urine, and BAL fluid, from mice infected with mucormycosis. Urine samples are preferred because of its higher sensitivity and small number of substances/materials impeding with PCR [120]. For the recognition of bacteria and yeast, Proteomic Profiling (MALDI-TOF) is performed although this is not well suited for the identification of fungi due to differences in growth rates and complex structures (Sendid et al., 2013; Lackner et al., 2014).

## Treatment of Mucormycosis

For successful treatment of any disease, cumulative strategies to combat the spread are required. For treating MCR, the multimodal approach that needs to be followed includes four major treatment-based strategies. Diagnosis of infection at early stages is the first and foremost step. Previous studies have highlighted the importance of early treatment in increasing the survival rate of the patients. Early diagnosis can therefore enable initiation of therapies with almost double efficiency and survival rate as compared to the same treatment done a few days later. There is further need to develop better diagnostic techniques for rapid detection of this invasive infection (Spellberg and Ibrahim, 2010). Another important strategy that has paved way for adjunctive therapies is reversing the underlying factors that weaken the host defense mechanism and lead to greater susceptibility of the host to the pathogen. Thus, introducing certain corrective and preventive measures can reduce the severity of infection spread.

Surgical removal of the tissues, which are affected by the spores of the Mucorales, becomes important component of the treatment. In certain situations, the antifungal agents are prevented from penetrating into the infected site due to lower availability of drug. Hence, in those circumstances; it becomes crucial to undergo tissue debridement *via* necrosis, or surgical removal (Hamilos et al., 2011). Reports have suggested increased rates of mortality in patients not undergoing surgical removals. In a previous research, it was observed that the patients who did not opt for surgical resection due to fear of operative risks, died within a period of 1 month after being diagnosed by this angioinvasive disease. On the other hand, almost 80% of those patients who underwent the surgery survived (Cho et al., 2019) Another important parameter to manage the effectiveness of MCR treatment is the optimal dose of drug administered for the antifungal therapy, since overdose can lead to further complications.

### Surgery

Mucormycosis is a disease that often progresses quickly and antifungal medication is typically insufficient to manage this illness. Antifungal susceptibilities vary widely across mucormycosis agents, and furthermore, some strains may be highly AmB resistant. In addition to this, thrombosis, tissue necrosis, and characteristic angio-invasion are all associated with poor antifungal drug penetration, thus preventing anti-infective medications from reaching the infection site. As a result, causative species could have high susceptibility to the anti-fungal therapeutic *in vitro* but the same anti-fungal agent might be completely ineffectual when tested *in vivo*. While antifungals may stop further spread and kill the pathogen, surgical intervention is essential to address tissue necrosis due to the infection (Spellberg et al., 2009).

Surgical debridement of necrotic and diseased tissue has to be addressed at the earliest. Since surgery is nearly bloodless, debridement usually proceeds promptly. In a logistic regression model, surgery was found to be an independent predictor of a favorable outcome between mucormycosis patients and those who received a combination of anti-fungal treatment and surgical management had a survival rate of 70% (328 of 470 patients; Roden et al., 2005). It may be beneficial in cases with localized infections such as for an isolated pulmonary lesion but it is not a viable option for disseminated mucormycosis cases or for infections of certain areas of the brain or lung parenchyma near major vessels. Plastic surgery should be utilized to fix disfigured body parts in circumstances where surgery is successful (Skiada et al., 2018). Surgery is especially effective in cases of soft-tissue infections and ROCM infections. Surgical removal of infected sinuses and debridement of retro-orbital region would help in preventing the spread of infection to the eye, thus leading to high recovery rates in ROCM > 85% (Toumi et al., 2012). Since surgery is often disfiguring, plastic surgical intervention should be utilized to fix disfigured body parts in circumstances where the surgery is successful to improve life quality of patients.

### Antifungal Therapy

Antifungal therapy is the most effective and preferred treatment for mucormycosis. It is achieved by administration of regular doses of clinically effective and approved drugs. There are no specified drugs for the mucor species yet, but certain classes of broad spectrum antifungal agents work effectively and are species dependent. For instance, amphotericin B is considered to be most active in its mechanism of fungicidal action, but it is ineffective against *Cunninghamella bertholletiae* and *Apophysomyces elegans*. However, certain strains of Mucorales have become resistant to these drugs (Salas et al., 2012). Other drugs like Posaconazole (PCZ) and ICZ (triazoles) categorized under azoles are also effective against mucors up to a certain extent (Perkhofer et al., 2009); whereas others including itraconazole and terbinafine are active only against a few strains and are not preferred for treatment of invasive mucormycosis.

### Primary Monotherapy for First Line Treatment

#### Polyenes

Administering moderate doses of the lipid polyenes is the most effective and primary treatment method for mucormycosis treatment. It includes the conventional amphotericin B (AmB) and its new alternative and liposomal amphotericin B (LAmB). Patients that opt for LAmB have survival rate of 67%, which is twice that of patients receiving AmB drug (Gleissner et al., 2009).

The antifungal behavior of a polyene is attributed to its binding to sterols like ergosterol responsible for providing structural rigidity to the fungal cell. This makes pores on the membrane after it is binding and thus, preventing the cell membrane from functioning normally. The limited activity of the cell membrane would further cause leakage of the fungal cell components, and hence the proliferation of fungal colonies would not take place. But enormous intake of these drugs can cause cell death due to increased toxicity. In order to combat this nephrotoxicity, liposomal preparations are used for prolonged therapies (Riley et al., 2016).

The advantage of using these lipid preparations is safer administration over longer durations at higher doses. These liposomal formulations are found to be less nephrotoxic when compared to classical AmB preparations. The retrospective studies have further proven that the amphotericin B lipid complex (ABLC) is substantially more efficient as a treatment option over AmB or LAmB for many patients, particularly the ones suffering from mucormycosis affecting central nervous system and from diabetic ketoacidosis; but for the cases concerning the rhino-cerebral regions, administration of ABLC remains an exception as it displayed a lower efficacy rate when compared to the LAmB or AmB (Reed et al., 2008).

Out of all the available drugs, the most commonly used is liposomal preparations of the LAmB as it has a good therapeutic index (Cornely et al., 2014). The drug’s responsiveness to antifungal agents is dependent on the site of infection and also on the host. The patients suffering from hematological malignancies and the ones undergoing SOT or HSCT find it difficult and problematic to respond to these agents (Roden et al., 2005).

The laboratory isolated *in vitro* preparations of *Cunninghamella* sp. have high minimum inhibitory concentration (MIC) values for AmB (Dannaoui, 2002). Studies done for non-aspergillus molds have reported less than ≤0.5 μg/ml of MIC levels for AmB (Lamoth et al., 2016). The experimental data from the research done on 524 clinical isolates showed ≥97.5% effective Epidemiologic Cut off Values (ECVs) for the AmB drug on various species: 2 μg/ml for *M*. *circinelloides* as well as for *L. corymbifera* (Espinel-Ingroff et al., 2015).

The exact polyene dose for this monotherapy treatment is not fixed; however, studies suggest that the optimal starting dose lies in the range of 5–7.5 mg/kg/day for both LAmB and ABLC. LAmB is generally used for children and adults with mild symptoms of the fungal infections. Researchers do not find any additional pharmacokinetic or clinical advantage of higher dose administration but there is evidence of administering 10 mg/kg/day of LAmB in patients with cases of osteoarticular involvement and CNS related mucormycosis to limit the penetration of polyene inside the brain tissues (Walsh et al., 2001). Electrolyte derangements and raised nephrotoxicity are associated with higher dose of the LAmB (Lanternier et al., 2015). Studies have proved the effectiveness of early AmB therapy in patients. Delayed therapy could result in increased mortality rates due to infection by almost 2-fold (Chamilos et al., 2008). Therefore, considering the host specificity, optimal drug administration is done for successful treatment.

#### Azoles and Triazoles

Triazoles are the most commonly used antifungal agents in clinical settings. These function by alteration of the ergosterol biosynthetic pathway through inhibition of 14-α demethylation of lanosterol. This further results in inhibition of membrane bound enzymes that are involved in cell wall synthesis. It affects membrane permeability of the fungal cell as ergosterol is replaced by 14-α-methyl sterols (Odds et al., 2003). PCZ and ICZ belong to the category of second generation triazoles and are active against Mucorales due to presence of α-O-methyl group. This group enhances drug spectrum and the drug works against *Aspergillus* and other fungal species (Cuenca-Estrella et al., 2006). There is absence of any reliable information on effectiveness of the azoles drugs like voriconazole (VCZ), fluconazole (FLZ), and itraconazole (ITZ) against the mucor species. The target of ITZ is specified to *Absidia sp*. only (Sun et al., 2002).

#### Posaconazole

This drug is structurally similar to ITZ and is considered to have a species dependent variable *in vitro* action against the causative agents of mucor related invasive infections (Dannaoui, 2002). Studies have proven that *Mucor* spp. is more responsive to PCZ. PCZ has been recommended by the European Conference on Leukemia Infections for the management therapy or in salvage therapy, whereas the guidelines proposed by the ECMM and the European Society for Clinical Microbiology and Infectious Diseases (ESCMID) recommend the use of PCZ as first line of treatment at moderate levels of administered oral suspension dose of about 200 mg especially for the patients who are non-responsive to LAmB. Due to its commercialization as tablets and intravenous injectable, the bioavailability and the exposure to the drug are increased (Wiederhold, 2015).

The exact role of tablets and intravenous IV formulations in patients suffering from mucormycosis is yet to be defined. The major reason due to which the need for the development of intravenous or gastro-resistant tablets was felt is the therapeutic failure in absorption of the drug from the oral suspensions which were mixed with high fat-based food or with acidic carbonated beverages (Dolton et al., 2012). Besides increased bioavailability, there are several benefits of taking tablets. These include improved pharmacokinetics; single daily dosage; increased resistance to drug motility, pH or gastric changes; no requirement for transferring medicine to patient; and reduced interpatient variability (Krishna et al., 2012). Despite all these properties, it is necessary for this drug to undergo the tests of Therapeutic Drug Monitoring (TDM).

Negative absorption of this drug is due to the presence of P450 enzymes hepatic cytochrome inducers, for instance rifampin (RMP) and phenytoin (PHT) that are mainly responsible for clearance of PCZ from our bodies. Other factors, which influence the absorption of this drug, are common clinical issues resulting from chemotherapy, immunosuppression, mucositis, and diarrhea (Dolton et al., 2014). According to a study, the required MIC after obtaining 1 to ≥4 μg/ml of dose comes out to be 90% (MIC90; Sun et al., 2002). The recommended dosage of this drug is 400 mg × 2/day when taken with meals. On the other hand, if not taken with meals, as the dosage can be 200 mg × 4/day. In cases such as when a patient is unable to take the oral form of the drug, a novel and excellent pharmacokinetic alternative of intravenous PCZ formulation with β-cyclodextrin has been produced (Cornely et al., 2014).

Spellberg et al. suggest against the use of oral PCZ as a primary drug due to variations in dose requirement as depicted in the pharmacodynamic and pharmacokinetic data concerning the reliability of this drug for treatment of mucormycosis for patients suffering from neutropenia, IFIs, or for the ones at a greater risk for malabsorption (Spellberg and Ibrahim, 2010).

#### Isavuconazole

Another triazole, which has been recently developed is isavuconazole (ISZ) and is considered to be a reliable alternative to AmB when taken at a moderate dose of 200 mg. This new azole has proven that its use for primary treatment in clinical trials is quite effective and therefore it has been approved for mucormycosis treatment in Europe and the United States and for cases wherein AmB is either not effective or not available (Marty et al., 2016). This drug is structurally similar to FLZ and is a wide spectrum azole proved to be effective against invasive mucormycosis. It is commercially available in the form of isavuconazonium sulfate prodrug, which gets activated by the serum butyryl cholinesterase. About 372 mg of this prodrug is considered to be equivalent to 200 mg of ICZ and needs to be administering every 8 h until sixth dose, followed by 372 mg/day (Riley et al., 2016). Similar to PCZ, it has *in vitro* species dependent variability but with an increased MIC level which is 2–4 times as high as that of former (Arendrup et al., 2015). This drug is available both as oral suspensions as well as intravenous formulations. It is administered at a moderate dose of 200 mg thrice per day (200 mg t.i.d) consecutively for first 3 days, thereafter followed by 200 mg. This azole has an advantage over other agents like lesser hepatotoxicity; no nephrotoxic cyclodextrin; less intra drug interactions; no skin or ocular side effects; QT prolongation; no requirement for meals; and excellent oral bioavailability. Hence, it does not require TDM (Trang et al., 2017).

Certain studies have reported the cases of prophylaxis after administering ICZ especially the ones in which patients were pre-exposed with trace amounts of AmB. Invasive fungal infections have been reported, especially mucormycosis among patients receiving this drug for treatment (Fung et al., 2018). Around 18% of the patients facing this problem had prior health issues like myeloid leukemia in retrospective trials (Cornely et al., 2015). A recent report highlights that 4.6% of the patients suffering from hematologic malignancies and undergoing ICZ therapy for almost a week contracted this infection (Dadwal et al., 2016).

### Combination Antifungal Therapy

For the immunocompromised patients, a combination of antifungal therapies is the treatment of choice. There exists lack of relevant clinical studies to prove the effectiveness of this therapy, yet this treatment option is being used with some pros and cons. It is a broad spectrum-based method having synergistic effect, but due to involvement of interaction of drugs, is claimed to be toxic and antagonistic (Spellberg and Ibrahim, 2010).

Research investigations have examined the *in vivo* and *in vitro* properties of the combined echinocandins and polyenes drugs. Echinoderms facilitate phagocytosis of the fungal agents through immune epitome unmasking resulting from its degraded action over the β-1, 3-glucan surfaced in minute traces on the fungal cell wall. This mechanism can be observed in the organisms specifically targeted by this drug (Ibrahim et al., 2008; Gebremariam et al., 2017).

For increased efficiency of therapeutics, echinocandins can be added to the backbone of polyenes (Ibrahim et al., 2008). Combined use of AMB and echinocandin is successful for patients suffering from rhino-orbital or cerebral mucormycosis due to underlying diabetes (Reed et al., 2008). But, on the other hand, for the patients suffering from malignancies, this treatment approach failed. However, the studies which combined polyenes with PCZ triazole, have displayed positive impact (Kyvernitakis et al., 2016). Patients suffering from hematologic disorders, who did not respond to primary mono-therapeutic drugs, displayed improvements in 56% of cases, when treated with combination therapy. It is essential to not exceed the dose more than 3 mg/kg/day especially against murine mycosis infections. The recently synthesized and approved monotherapeutic drugs, when combined with caspofungin (CAS) show remarkable synergy with AmB and triazoles (Guembe et al., 2007). Combination of azoles with echinocandin does not exhibit any therapeutic synergy (Gebremariam et al., 2017). Enhanced fungal clearance and improved survival rates have been reported from the *in vivo* studies of LAmB combined to echinocandins; whereas ABLC + echinocandins only are helpful for decreasing mortality rates (Spellberg et al., 2005). Details on recommended anti-fungal agents along with their modes of action, doses and duration are given in Table 2.

**Table 2:** Recommended antifungal agents and doses.

S. no.	Drug	Mode of action	Recommended dose	Duration
1.	Amphotericin B (AmB) Liposomal Amphotericin B (LAmB) Amphotericin B Lipid Complex (ABCL)	Pore formation on fungal cell membrane on binding to ergosterol and ROS production.	5 mg/ml/day With CNS complications: 10 mg/ml/day	6–12 weeks
2.	Posaconazole (PCZ)	Inhibiting lanosterol 14α demethylase, thus preventing ergosterol production	Oral supplements: 4 × 200 or 2 × 400 mg/day Tablets/IV formulation: 2 × 300 mg/day 1 followed by 1 × 300 mg/day	6 months
3.	Isavuconazole (ICZ)	Inhibiting the lanosterol 14α demethylase	Orally/IV formulation: 3 × 200 mg/day 1 × 200 mg/day	3 months
4.	Combination	Echinocandins act on fungal cell wall and inhibit the β-1, 3-glucan synthesis	LAmB + PCZ/ICZ LAmB + echinocandin	3–6 months

**Figure 4:**
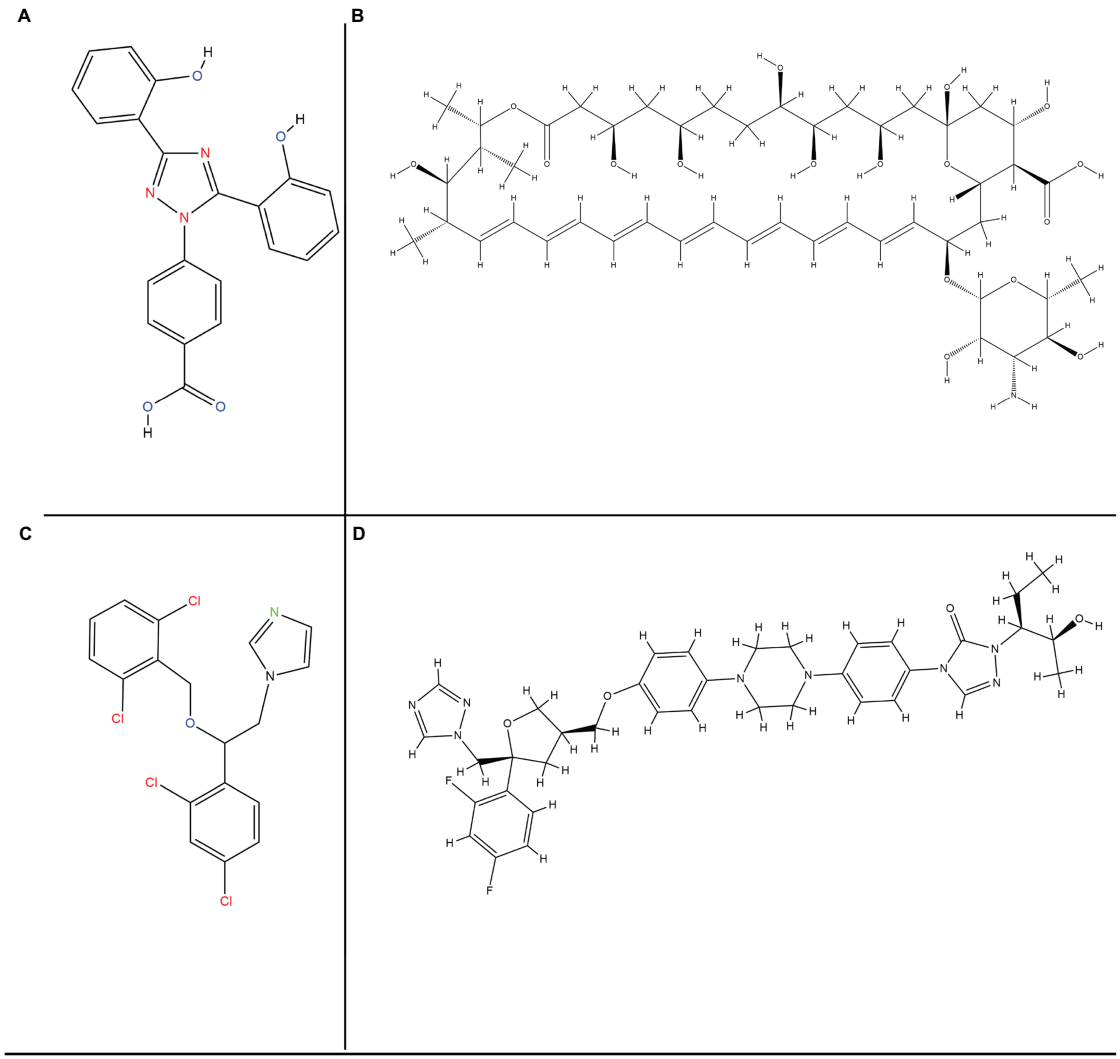
Chemical structure representations of various drugs used for treatment approaches **(A)** Deferasirox, **(B)** Amphotericin-B, **(C)** Isavuconazole, and **(D)** Posaconazole.

Non-antifungal agents like the iron chelators displayed good synergy when combined with antifungal drugs, but their effectiveness in patients is yet to be proven (Reed et al., 2008). Certain calcineurin immunosuppressive inhibitors like tacrolimus and cyclosporin A have demonstrated modest synergy with antifungal medications *in vivo* and *in vitro* (Schwarz et al., 2019). A Hos2 histone deacetylase inhibitor, MGCD290 is another agent that has displayed *in vitro* synergy with drugs like PCZ and (VCZ), but these have not yet been approved by FDA (Chamilos et al., 2008). *In vitro* studies have displayed more than 50% synergic effects of two more combinations of RMP with AmB and azoles along with miltefosine (Biswas et al., 2013). It must be considered that different species react differently to these drugs. Moreover, data available for this therapeutic technique are contradictory. Hence, this method of treatment should be preferred only when the patient shows no response to primary monotherapy or has a less success rate. Structural information of various drugs used for the treatment is depicted in Figure 4.

### Salvage Therapy

The patients intolerant to the primary treatment are advised to undergo salvage therapy of PCZ and deferasirox (DFS; Spellberg and Ibrahim, 2010). The foundation of salvage therapy had been laid by the combination of drugs. Even though PCZ persists in the host even after several months of administration, it is considered to be the safest drug for salvage therapy. The success rate for PCZ drug used for salvage therapy as oral suspension of four divided doses of 200 mg was about 70% in a trial, but some of the patients complained of gastrointestinal problems (Greenberg et al., 2006).

The response rate of the drug in patients with refractory mucormycosis is 61%; and among patients with pulmonary mucormycosis, it is as much as 65%. Also, it was found that at the end of 12 weeks of undergoing PCZ salvage therapy, 21% of the patients were stabilized (van Burik et al., 2006). Since there is a risk of increased DFS toxicity after 4 months of therapy, it is advisable to undergo salvage treatment with DFS for 2–4 weeks only. But the dosage should be 20 mg/kg/day for not more than 4 weeks. Periodic monitoring of hepatic and renal functioning must be done to ensure there are not associated side effects (Nick et al., 2002).

The sixth European Conference on Infections in Leukemia (ECIL-6) has proposed using LAmB either with CAS or PCZ, since both of them cannot be successfully used in first line treatment (Kyvernitakis et al., 2016). Some evidence has supported the use of ICZ as the salvage therapy option to treat mucormycosis infection.

### Adjunctive Therapies

#### Immune Augmentation Therapy

Previous research has suggested granulocyte activity effectively kills fungal pathogens responsible for mucormycosis due to the action of colony stimulating factors (CSF), interferon-γ (IFN-γ), and pro-inflammatory cytokines (Gil-Lamaignere et al., 2005). However, the role of these recombinant cytokines is not well defined for primary treatment but some case reports support immune therapies using these factors as adjunctive treatment. But these have also been associated with inflammatory lung injuries (Mastroianni, 2004). For patients suffering from neutropenia based mucormycosis, these granulocyte-based transfusions have proved to be effective to a great extent.

A severely immunocompromised patient within tractable mucormycosis was treated successfully by combining IFN-γ with nivolumab (Grimaldi et al., 2017). IFN-γ is believed to restore monocyte function, whereas nivolumab is a monoclonal Ab that reduces Programmed cell death protein 1 (PD-1) expression of T-lymphocytes by blocking its ligand interactions (Gamaletsou et al., 2012).

#### Immunosuppression Reversal Therapies

Since immunosuppression reversal is significant for a successful treatment besides early diagnosis and surgeries; many patients suffering from this infection are found to have poor bone marrow recovery and are in need of prolonged immunosuppression therapies, especially in patients who have GVHD or neutropenia. They need to undergo adjunctive immunotherapy supplemented with hematopoietic growth factors or white cell transfusions so as to reverse the effects of all the underlying problems. In patients who have been immunocompromised due to excessive use of steroids could undergo non-steroidal therapies. Those suffering from AIDS can be given antiviral therapies to increase immunity. Hyperglycemia or excessive sugar intake needs to be avoided by diabetic or ketoacidotic patients. They must also undergo sodium bicarbonate based acidemia reversal to partially obstruct the *R. oryzae* attack over endothelial cells and hence, restoration of iron chelation of host and the lost neutrophil action (Gebremariam et al., 2016).

#### Iron Chelation Therapy

**Figure 5:**
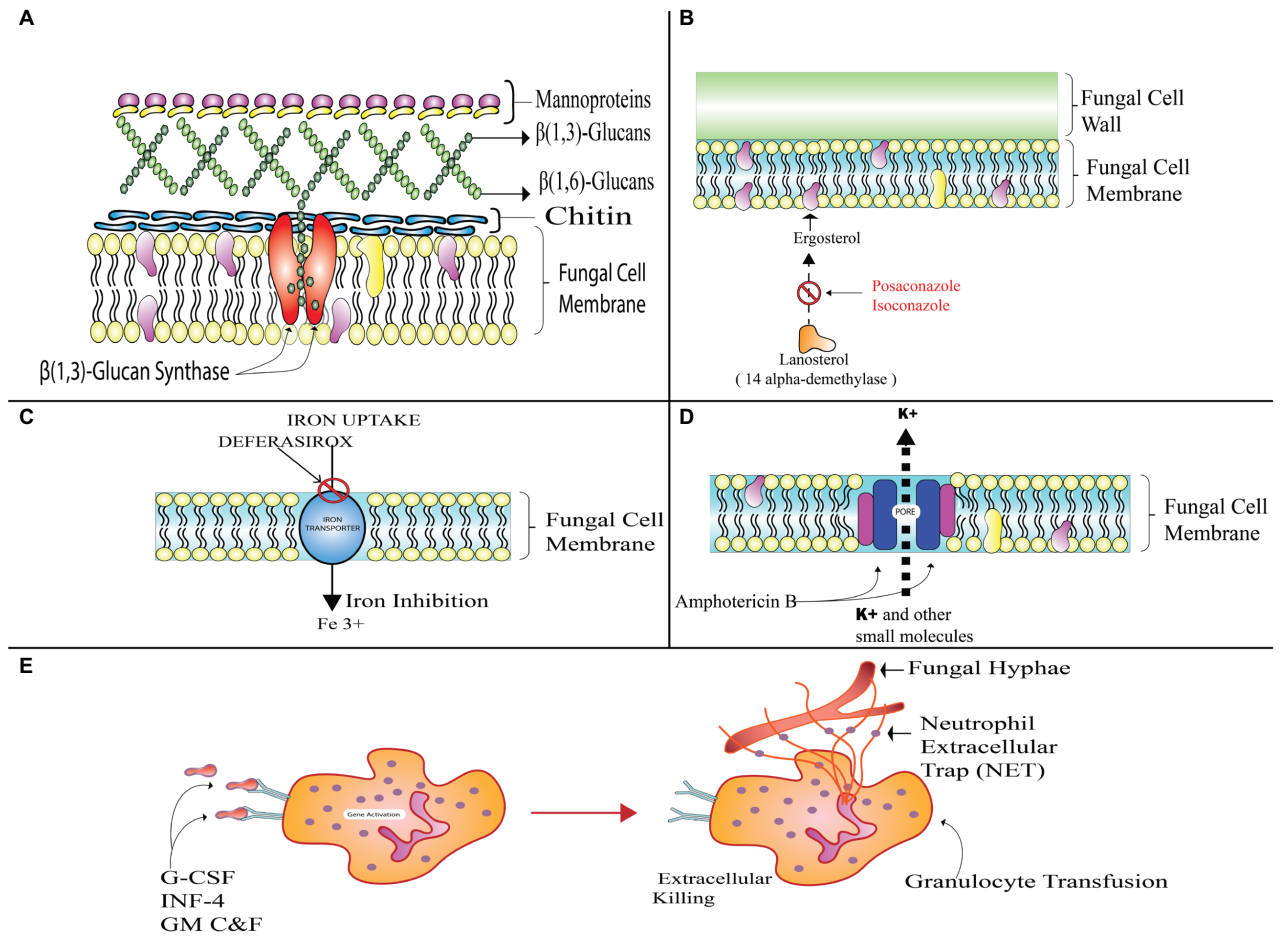
Drugs used for treating mucormycosis- targets and modes of action. **(A)** Represents the pore formation on fungal membrane after binding of polyene with ergosterol; **(B)** Triazole mediated inhibition of ergosterol synthesis by inhibition of lanosterol 14-alpha- demethylase enzyme; **(C)** Echinocandins halt the beta-glucan linking of cell wall; and **(D)** Iron chelation therapy by deferasirox (DFS) inhibiting fungal uptake of iron. **(E)** Cytokine therapy activating granulocyte transfusion can aid treatment by damaging fungal cell.

The importance of iron for host immune system is widely known, thus, iron chelation therapy is one of the potential approaches for adjunctive treatment. It works by reduction in the iron concentration to halt fungal growth. The most common iron chelator in clinical use is DFS, which enhances patient survival and lacks siderophore capability. Fungicidal activity of this chelator was observed at 6.5 mg/ml dose with MIC 90 (Ibrahim et al., 2007). It has a time dependent action that gives results in 12–24 h after exposure. Molecular target and modes of action of drugs used for treating mucormycosis are presented in Figure 5.

In a randomized study on patients suffering from various malignancies, high mortality was noted on treatment with a combination of DFS and LAmB, as DFS enhances the rate of iron delivery to the Mucorales species (Spellberg and Ibrahim, 2010). However, this therapy has proved to be beneficial for patients with diabetes and ketoacidosis as the underlying disease condition with 80% survival rate (Cornely et al., 2014). Iron chelation therapy from orally administered DFS was approved for treating iron overload by US FDA in the year 2005, for the patients having transfusion-dependent anemia (Cappellini, 2005).

Deferasirox can be successfully used for salvage therapy among the mucormycosis patients suffering from complications in rhino cerebral regions having progressive brainstem related disease (Reed et al., 2006). There are multiple limitations of this therapy especially when it comes to absorption failure. Due to its availability as oral supplements, the patients undergoing intestinal surgeries must avoid this drug in particular. Gastrointestinal complications like diarrhea and nausea are most likely to happen after administration of DFS. Due to elevated creatinine levels in patients receiving this drug, the major concern is renal toxicity, causing hemodialysis and ultimately leading to renal failure (Vichinsky, 2008).

## Role of Nanomedicine

The role of nanotechnology in the arena of drug delivery is very significant, crucial, and beneficial. Modern day diseases are occurring at a higher rate and the available drugs are not much effective compared to the prevalence of the infection. Moreover, antimicrobial resistance has made it much more difficult to manage the upsurge of diseases. Hence, the role of novel agents like nanoparticles (NPs) comes to the fore. These are supramolecular ultra-dispersed particles, which lie in the size range of 10–1,000 μm. These minute particles have benefits in targeted drug delivery, facilitating greater bioavailability, enhanced antifungal properties, reduced toxicity, and target specificity. The various delivery mechanisms include carrier-based methods like solid and nanostructure lipids, dendrimers, liposomal preparations, and polymeric NPs (Voltan et al., 2016).

**Figure 6:**
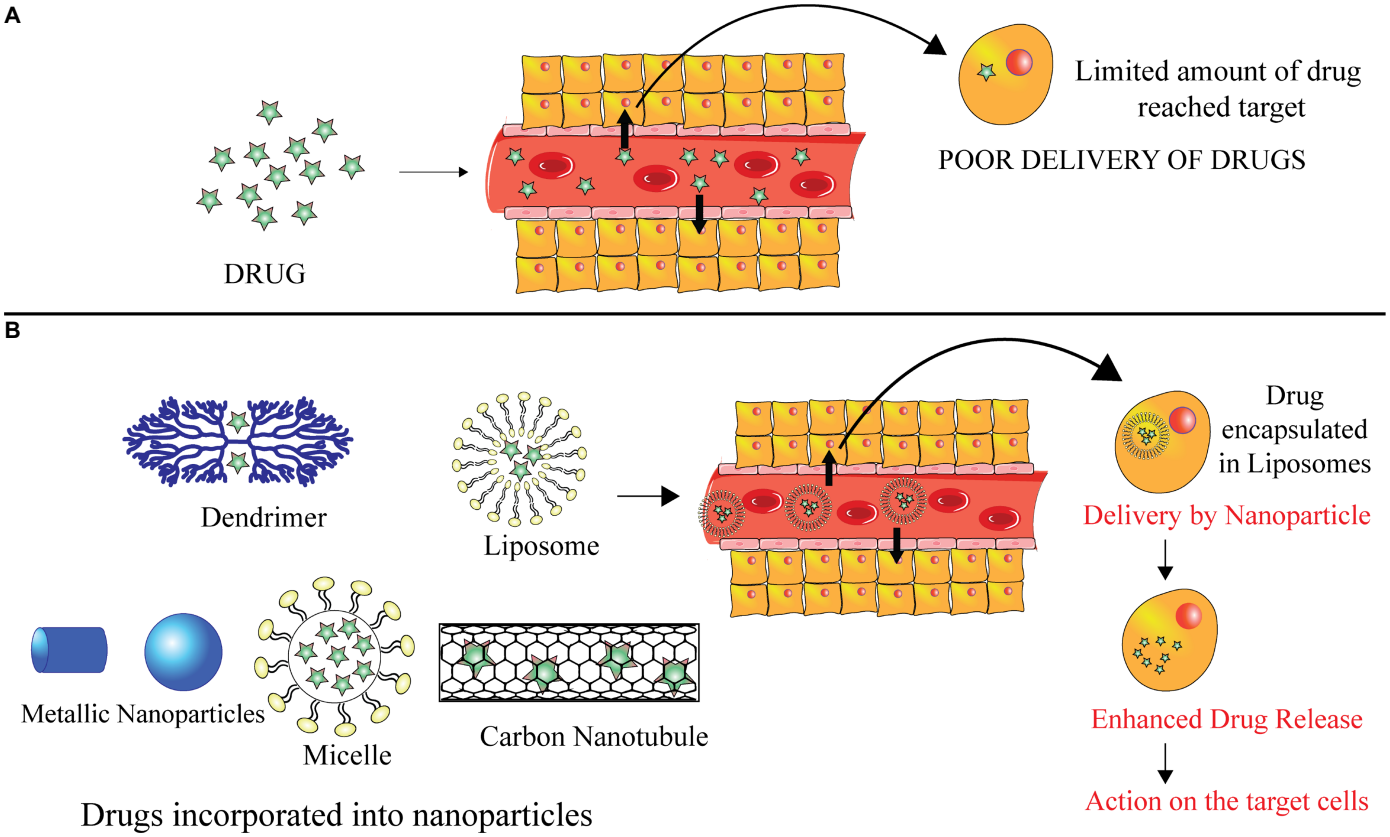
Drug delivery mechanisms **(A)**. Conventional drug delivery targeting the affected region and displaying poor incorporation inside the tissue **(B)**. Drug delivery with the help of nanoparticles like dendrimers, carbon nanotubes, SNPs, micelles, and liposomes. These help in better and enhanced delivery of drug to the affected tissues and cells.

These drug delivery mechanisms are being utilized at a larger scale for clinical applications. A drug could be entrapped, bound to the NPs matrix, dissolved or encapsulated (Calixto et al., 2014). During earlier times, intravenous administration of pharmaceutical suspension was a cause of concern as it led to embolism. However, presently, nano-pharmaceuticals and nanomedicine have paved way for improved technologies for diagnosis and treatment and hence, in combating fungal infections to a greater extent with better efficiency. The treatment procedures that are fundamentally based on the delivery mechanisms are crucial to maintain drug concentration and toxicity levels (Couvreur, 2013). The reduced size of the carrier further helps to target specific cells and tissues and has minimal side effects. The factors responsible for the efficient delivery of the drugs using nanotechnology include intrinsic chemistry of NPs, their hydrodynamic size, administration route, shape, quantity, and circulation duration inside host and its reactivity with the host immune response (Weissig et al., 2014). The lipid formulation of drugs like AmB or nystatin can be combined to reduce the toxicity of the conventional drugs. SNLs and Nanostructured lipid carriers (NLCs) based delivery systems have been tested for antifungal drugs. Another form of lipid suspensions called liposomes is composed of layers of phospholipids. These are capable of mobilization of hydrophilic drugs inside their aqueous core. They have an advantage of increased penetration and protect the drug from degradation besides being biocompatible (Hunstad et al., 1999). Polymeric Nanoparticles (PNPs) were also used but due to polymer degradation and higher costs are replaced by Solid Nanoparticles (SLNs) colloids due to their physical stability and different administration routes (Pardeshi et al., 2012). Newer SLNs are known as NLCs and polymer lipid hybrid nanoparticles (PLNs). These are the conjugates of solid-lipid matrices that entrap the drugs inside their compartments and possess greater drug loading capacities (Müller et al., 2002). Another class of drug delivery systems includes dendrimers that are structured into core, dendrons, and surface-active groups from inside to outside. Antifungal activity of these dendrimers is attributed to their ability to inhibit candida 1, 3-β-d-glucan synthase enzymes and therefore, change the morphology of fungal cells (Janiszewska et al., 2012). Reports have also suggested the role of other delivery systems displaying antifungal activity, for instance magnetic nanoparticles (MNPs), carbon nanotubes, and silica NPs (Voltan et al., 2016). As per latest research, silver nanoparticles (AgNPs), due to the presence of β-cyclodextrin (George et al., 2011); Zirconium oxide nanoparticles (ZrO_2_NPs; León-Buitimea et al., 2021), and nano-emulsion NB-201, due to presence of benzalkonium chloride (BZK; Brunet and Rammaert, 2020; Kajfasz et al., 2020), exhibit antifungal properties with higher toxicity against Mucorales and relatively low toxicity in human cells. Detailed mechanism of incorporation of drugs in nanoparticles is provided in Figure 6.

## Novel Treatment Approaches

Apart from the drugs in use for primary mono-therapeutic techniques, novel treatment strategies specifically concerning the host and fungal pathogens are being continuously worked upon. An approach to discover new administration routes for aerosols to enhance the treatment procedure is also under development stages (Brunet and Rammaert, 2020). Such agents are gaining momentum due to their pharmacological significance over the already available drugs. The use of newer approaches like nanotechnology and antifungal microbial peptides to treat and manage mucor infections is the future of mucormycosis treatment.

### New Antifungal Drugs

Various antifungal agents are currently under clinical trials. Some of these include SCY-078 (MK-3118), encapsulated AmB, rezafungin, and orolofin (Van Daele et al., 2019). MK-3118 or SCY-38 belongs to the subfamily of a new glucan class synthase inhibitor. It has been found to be poorly effective against Mucorales species (Lamoth and Alexander, 2015).

Another form of AmB, the encapsulated form is an oral formulation of AmB. It has shown great tolerance in the studies carried on *Cryptococcus neoformans*; however, there is no information available on the efficiency of this drug against mucormycosis. A new echinocandin, i.e., Rezafungin is also not yet tested for Mucorales. The resistance of these fungal species to many antifungal agents is due to the genetic plasticity offered by the whole genome duplication that occurred in the process of their evolution as revealed by the genome sequencing of the *R. oryzae* (Kyvernitakis et al., 2016).

The antifungal drug VT-1161 possesses specific inhibitory action on the fungal CYP51 gene and is shown to be active against Mucorales *in vitro*. It is also in development stage. Studies have shown that this drug can be effectively used for prophylactic and curative treatment in mice models of *R. arrhizus*, *Lichtheimia* sp., and *Cunninghamella sp.* that show neutropenia. This drug prolonged the survival rates among those models (Gebremariam et al., 2017). A new triazole SCH42427, due to its wide spectrum of activity is found to be effective in murine models. APX001A, earlier referred to asE1210 is an agent that targets a surface protein Gwt1 involved in the glycosylphosphatidylinositol pathway of posttranslational modifications. It is under phase 1 clinical trials and its effectiveness is more than that of AmB (Rivero-Menendez et al., 2019). This drug also acts as a prodrug for manogepix (MGX) whose mechanism of action is similar to fosmanogepix (APX001); and is very compelling for the treatment for pulmonary mucormycosis. The US FDA has recently approved oral and intravenous formulations of this drug for treating certain fungal infections.

Another azole, PC1244 displayed its efficacy against mucorales with MIC in the range of 0.25–2 mg/ml (Colley et al., 2018). Clinical analysis of an antibiotic drug colistin has proven it to be active against mucorales (Ben-Ami et al., 2010). Recently, scientists created a new therapy that uses the anti-CotH3 antibodies synergic with the antifungals and used it for prevention of mucormycosis in mice suffering from neutropenia and diabetes. These antibodies prevent the endothelial invasion by blocking the GRP78 binding with CotH3 peptide (Gebremariam et al., 2019).

Another novel agent, haemofungin has been found to inhibit fungal growth *in vitro* (Ben Yaakov et al., 2016). Fluvastatin is another drug that belongs to the category of statins and has fungicidal action targeting a wide range of fungal species including Mucorales (Tavakkoli et al., 2020).

Glucocorticoids like dexamethasone and methylprednisolone have been in use for the treatment of SARS-CoV-2 patients due to their potential to curb inflammation. These are anti-inflammatory and immunosuppressive in action and may thus prove to be beneficial for mucormycosis patients. Some natural compounds like luteolin, colchicine, allicin, resveratrol, curcumin etc. obtained from plant sources are also being investigated for their inhibitory activities against chemokines and cytokines. These works by targeting signaling pathways such as MAPK/ERK, NF-κB are involved in the production of chemokines and cytokines (Peter et al., 2020). Further, the role of mesenchymal stem cells (MSCs) in the reducing the effects of cytokine storm induced due to COVID-19 has also been examined (Wang et al., 2020).

### Antifungal Microbial Peptides

Many therapeutic candidates like monoclonal antibodies (mABs), immunomodulatory molecules, checkpoint inhibitors, and antimicrobial peptides (AMPs) have been studied to possess antifungal properties. A study by Barbu and coworkers showed the effects of antimicrobial peptidomimetic motif D (KLAKLAK) on Mucorales leading to killing of Mucorales through mitochondria-mediated apoptosis. The properties attributed to this fungicidal action are the inhibition of fungal germination due to reduced hyphal viability. The researchers found a direct relation of the antifungal property of these agents with many factors, including depolarization of mitochondrial membrane, vacuolar injuries, intracellular ROS species, mitochondrial swelling, and enzymatic action. Post-antifungal effects were also limited and it was concluded that this potential agent causes death of hyphae due to depolarization of plasma membrane as well as spores. The cell apoptosis is a result of the mitochondrial injury and provides insights into further development of potential drugs, therapeutics and fungicides (Barbu et al., 2013).

By targeting cells and stimulating complement activation and phagocytosis, many fungal-specific mABs can be utilized to treat invasive fungal infections, including black fungus (Casadevall and Pirofski, 2012). Synthetic AMPs have been in use in antifungal therapies; these have membrane lytic activity against fungal species. Certain species of AMPs have different mechanisms of action like toroidal pore formation, membrane curvatures induced by peptides, thickening or thinning of membranes, flip-flop of membrane, lipid clustering, or disrupting potential of membrane. The antifungal peptides in clinical and preclinical stages of development include NP213, CZEN-002, P113, HPX-124, hLF-11, omiganan, LTX-109, Isoganan (IB-367); NP399, D2A21, and EDT-151. All these exhibit some fungicidal properties (Mercer and O'Neil, 2020), which could be utilized as therapeutic alternatives for treatment of mucormycosis.

The factors hindering the development of these new techniques include the inappropriate testing procedures. Hence, these cannot be developed into drugs since data for the duration, doses, administration route, and formulations have not been optimized until now. But it is accepted widely that these AMPs are potential candidates for treatment of mucormycosis. The fungicidal properties of these compounds can be utilized in the form of commercialized products.

## Conclusion and Future Perspectives

The causative agents of black fungus are the opportunistic pathogens that particularly target the immunocompromised high-risk patients. These pathogens grow and colonize after finding suitable conditions and consequently, amplify the disease to such an extent that if not treated timely, it proves to be fatal. The trilogy of SARS-CoV-2, DM, and prolonged hospital stay are the major culprits for the rapid upsurge in Mucormycosis infections worldwide.

There are certain limitations to the currently available antifungal therapies and the diagnosis techniques that make it necessary to develop methods and techniques which are comparatively more efficient than the conventionally available techniques. The fact that early diagnosis can help to eradicate this infection cannot be overruled. Developing effective diagnostic procedures is critical so that therapeutic procedures can be initiated at disease onset and spread of the infection to other tissues can be forestalled. Although devising an accurate detection method is a challenge, various molecular techniques have paved way for faster analysis. More rapid techniques including the metabolic and serological tests that do not require invasive protocols are being worked upon for better and faster detection (Skiada et al., 2020). Mucorales are generally resistant to antifungal agents. Moreover, the data for antifungal susceptibility and MIC values for Mucorales are limited (Sipsas et al., 2018). This considerably limits the choices of antifungal agents and the success of monotherapies. One of the major issues faced using drugs-based treatment methods is the inefficient delivery of drugs to the affected tissues. The conventional drugs those in use also have limited efficacy, less selectively and very poor biodistribution, which can be overcome by shifting to relatively recent NPs based drug delivery mechanisms. But the disadvantage of using nanotechnology based solutions is greater production costs. However, nanotechnology-based approaches aid in better delivery of drugs, peptides and muco-adhesive systems with enhanced retention capacities and improved specificity can be achieved (Voltan et al., 2016).

These alternative solutions need to be worked upon for better management, increased bioavailability, and treatment of invasive infections, especially when the specified narrow spectrum drug is not available. The novel antifungal agents in various developmental phases are promising candidates for combating the invasive growth of the fungal species responsible for this deadly disease. A way forward can be to test decolonization of spores while they are in dormant phase (Brunet and Rammaert, 2020). With development of antimicrobial peptides, their proven fungicidal property can be exploited in future to develop better treatment options.

## Author Contributions

All authors listed have made a substantial, direct and intellectual contribution to the work, and approved it for publication.

## Conflict of Interest

The authors declare that the research was conducted in the absence of any commercial or financial relationships that could be construed as a potential conflict of interest.

## Publisher’s Note

All claims expressed in this article are solely those of the authors and do not necessarily represent those of their affiliated organizations, or those of the publisher, the editors and the reviewers. Any product that may be evaluated in this article, or claim that may be made by its manufacturer, is not guaranteed or endorsed by the publisher.

## Abbreviations

ROCM: Rhino-orbito-cerebral mucormycosis
HSCT: Hematopoietic stem-cell transplant surgeries
IFIs: Invasive fungal infections
DM: Diabetes mellitus
DKA: Diabetic ketoacidosis
HM: Hematological malignancies
CAM: COVID-19 associated mucormycosis
CDC: Centers for Disease Control and Prevention
CT: Computed tomography
MRI: Magnetic resonance imaging
ITS: Internal transcribed spacer
AmB: Amphotericin B
ABLC: Amphotericin B lipid complex
PCZ: Posaconazole
ECMM: European confederation of medical mycology
ISZ: Isavuconazole
DFS: Deferasirox
CSF: Colony stimulating factors
NLCs: Nanostructured lipid carriers
