# Supplementary material for: Mucormycosis Amid COVID-19 Crisis: Pathogenesis, Diagnosis, and Novel Treatment Strategies to Combat the Spread
Source: Front Microbiol. 2022 Jan 4;12:794176. doi: 10.3389/fmicb.2021.794176 (PMC8763841; doi:10.3389/fmicb.2021.794176)
Supplement: Supplementary file 1 [file Data_Sheet_1.DOCX]

**Supplementary figure 1:** **(a-c)** depict zygospores with various suspensors in different species; figure **(d-e)** shows different sporangiophore morphology; figure **(f)** shows columella of *Backusella* *recurva* **(g-i)** show morphology of sporangium at different stages of its life cycle; **(j)** depicts the sporangiophore along with the columellae and **(k)** hyphae of *Rhizopus microsporus* in human lung tissue (1).

**Supplementary figure 2: a**) Sporangiophore with indistinct apophysis and (**b**) columella and detached sporangiospores of *Rhizopus arrhizus*; (**c**) branched sporangiophore of *Mucor circinelloides*; (**d**) branched sporangiophore of *Mucor ramosissimus*; (**e**) sporangiophore with columellae and detached sporangiospores of *Lichtheimia ramosa*; (**f**) sporangiophore with columellae and detached sporangiospores of *Rhizomucor pusillus*; (**g**,**k**) sporangiophores of *Apophysomyces variabilis*; (**h**) sporophore with sporangiola and columella; (**i**) sporophore of *Saksenaea sp.*; (**j**) sporophore with merosporangia of *Syncephalastrum racemosum*; (**l**) young sporangiophore of *Actinomucor elegans (****m****) sporophores with single-spored sporangiola of Cunninghamella bertholletiae (1)*

**Supplementary table 1**: Approximate MIC values of different drugs used against 5 species causing Mucormycosis (2).

| **Drugs** | **Amphotericin (AmB)** | **Itraconazole (ITZ)** | **Posaconazole (PCZ)** | **Voriconazole (VCZ)** | **Isavucanozole (ISZ)** |
| --- | --- | --- | --- | --- | --- |
| **Species** |  |  |  |  |  |
| *Lichtheimia corymbifera* **Range** | 0.03-10 | 0.03-2 | 0.06-2 | 2->16 | 0.125->16 |
| MIC 50 | 0.25 | 0.25 | 0.25 | >16 | 4 |
| MIC90 | 0.5 | 1 | 1 | >16 | >16 |
| *Mucor* *sp****.* Range** | 0.03-8 | 0.03->16 | 0.03->16 | 0.125->16 | 4->16 |
| MIC50 | 0.25 | 2 | 1 | >16 | >16 |
| MIC90 | 0.5 | >16 | >16 | >16 | >16 |
| *Rhizopus arrhizus* ***Range*** | 0.06-2 | 0.25->16 | 0.125->16 | 2->16 | 1->16 |
| MIC50 | 0.25 | 2 | 0.5 | 8 | 2 |
| MIC90 | 0.5 | >16 | 8 | >16 | >16 |
| *Rhizopus microspores* **Range** | 0.06-2 | 0.25->16 | 0.06->16 | 2->16 | 1->16 |
| MIC50 | 0.5 | 1 | 0.5 | 8 | 2 |
| MIC90 | 1 | >16 | 4 | >16 | 8 |
| *Rhizomucor pusillus* **Range** | 0.03-0.5 | 0.03->16 | 0.06-4 | 0.0125->16 | 4-8 |
| MIC50 | 0.25 | 0.5 | 0.5 | >16 | NA |
| MIC90 | 0.5 | 2 | 1 | >16 | NA |

**Anti-fungal drugs used for the treatment of Mucormycosis**

Broth microdilution method prescribed by the Clinical and Laboratory Standards Institute (CLSI) has been in use to determine the minimum inhibitory concentrations (MICs) and the drug concentrations required to inhibit 50% (MIC50) or 90% of pathogenic isolates (MIC90) like *Lichtheimia corymbifera, Mucor spp. Rhizopus arrhizus, R. microsporus and Rhizomucor pusillus* for AmB, ITZ, PCZ, VCZ and ISZ (2). Antifungal susceptibility testing should, in theory, yield a result that may be used to foretell the chance of an infection by that organism being treated successfully or unsuccessfully with the antifungal drug under consideration. MICs are a worthy measure of an antifungal agent’s *in vitro* potency. However, predicting the actual treatment outcome requires determination of additional parameters such as the Epidemiological Cut-Off Values (ECVs), Clinical Breakpoints (CBPs).

**Amphotericin B (AmB)**

AmB, among all the antifungal medications available, has the best *in vitro* action against most of the mucormycosis causing species as evident by the low MICs (as shown in Supplementary table 1) but the susceptibilities vary across species and different species correspond to different MICs.

Although AmB is effective, due to its toxicity and infusion related side effects, advanced formulations such as liposomal AmB are the preferable alternatives due to their higher efficacy and better tolerance. When lipid formulations of AmB, mainly liposomal AmB, were administered as first-line or salvage therapy, a number of retrospective studies revealed an increase in survival rates and several studies and case reports have also been published that describe successful treatment with this medication (3-6).

**Itraconazole (ITZ)**

Despite the fact that ITZ has not been used as first-line therapy for infections caused by Mucorales members, it has structural and pharmacological similarities with PCZ. As per *in vitro* studies, ITZ is more effective against Zygomycetes than VCZ, but the MICs vary according to species and some selective species with low MICs might be highly susceptible to ITZ. As a result, ITZ may be beneficial in some cases of mucormycosis involving susceptible strains (2).

**Posaconazole (PCZ)**

In the azole drug family, PCZ is the first drug to exhibit wide spread action against Mucorales and furthermore the *in vitro* investigations have established its efficacy against these pathogenic fungi (MIC50 ≤ 1 mg/L). Some species are highly susceptible to PCZ and show significant inhibitory action, specifically members of the family Lichtheimiaceae (*R. pusillus* and *L. corymbifera*) as evident by their low MIC values. Furthermore, in vivo action of this medication has been demonstrated in experimental models of infection. In mice infected with *Mucor sp*., PCZ treatment resulted in an increase in survival (7).

**Voriconazole (VCZ)**

Despite the fact that VCZ has extremely poor efficacy against Mucorales, it has been used for comparative analysis due to its structural similarity with ISZ (8). As is evident by MICs; VCZ *in vitro* has almost no effect on the Mucorales. MICs >8 mg/L have been noted for this drug, thus proving it is not much effective against these fungal pathogens.

**Isavuconazole (ISZ)**

ISZ, unlike VCZ, has detectable but modest *in vitro* inhibitory activity against some Mucorales species. Although considering that the drug exposures *in vivo* are frequently greater with ISZ than with PCZ, it is unclear if the reported MIC differences between PCZ and ISZ with Mucorales members are clinically relevant (9-11).

**Impact of SARS-CoV-2 on the immune system**

SARS-CoV-2 triggers both the innate and the acquired immune response in the host. Viruses trigger the release of inflammatory factors, the activation and maturation of dendritic cells (DCs), and an increase in the synthesis of type I interferons (IFNs), all of which are critical in restricting viral propagation (12). B cells are stimulated by CD4+ T cells to secrete viral specific antibodies such as Immunoglobulins IgG and IgM whereas the CD8+ T cells directly kill the viral pathogens. Along with this, various pro-inflammatory cytokines and mediators are produced by helper T cells to aid the other immune cells in contrast to this, SARS-CoV-2 can thwart the immune system of the host by inhibiting T cell functions and triggering programmed cell death by apoptosis.

Patients suffering from severe COVID-19 show critical reduction in CD4+ and CD8+ T cells along with lymphopenia, lymphocyte dysfunction and an increase in neutrophils circulating in the blood with advent of circulating neutrophil precursors and a characteristic dysfunction of classical monocytes along with reduction of non-classical monocytes, furthermore the decreased abundance of dendritic cells and natural killer cells (13, 14). The levels of systemic inflammatory cytokines, particularly interleukin-6 (IL-6) and interleukin-1 (IL-1) are elevated (15). The levels of immunoglobulin G (IgG) and total antibodies are higher and in contrast the interferon level action is slower and attenuated (16, 17), this along with a plethora of complications such as hyperglycaemia, diabetic ketoacidosis, increased blood ferritin and immunosuppression due to overuse of steroids paves the way for increased risk of developing severe and in some cases potentially fatal mucormycosis infection.

**Immunosuppressants**

Steroids are used to fight the inflammatory response due to COVID-19. These corticosteroids hinder the body’s ability to fight infection by suppressing macrophages and neutrophils (18). Steroids diminish inflammation and immune system activity, lowering the generation of white blood cells (WBCs) and T-helper cells, making it easier for foreign substances to infiltrate and destroy the immune system in the host cell. These steroids may also cause an uncontrolled release of sugar, allowing the Mucorales to proliferate, reproduce, and invade at an accelerated rate.

**Diabetes/hyperglycaemia**

Reduced viral clearance, decreased T-cell activity, elevated cytokine storm, and immune-suppression are all plausible mechanisms by which diabetes enhances COVID-19 morbidity and mortality. (19) In COVID-19 patients, hyperglycaemia exacerbates the cytokine storm by disrupting endothelial cells, resulting in multi-organ damage. Mucorales thrive in diabetic ketoacidosis because of the acidic environment and increased quantities of free ferric ions. These conditions further favour the invasion and effective attachment of Mucorales hyphae inside the body.

The chemotactic factors generated by neutrophils are further diminished in patients undergoing excessive steroid therapy or in diabetic patients due to ketoacidosis or hyperglycaemic circumstances, resulting in an increase in fungal hyphae in human hosts (20).

**Iron**

Iron is abundantly released in immunocompromised individuals via sequestering proteins, creating a favourable environment and allowing the fungus to thrive inside the human body. Mucorales also use a high-affinity iron permease to uptake iron and deliver it to the host cell for growth (21). Fungi are frequently eliminated by polymorphonuclear phagocytes in healthy humans. As per the studies, the animals or humans with a reduced number of phagocytes or impaired phagocytic activity are more susceptible to mucormycosis infection. As a result, people with chronic neutropenia are more susceptible to contract the infection (22). Conversely because HIV/AIDS patients do not appear to be at a higher risk of mucormycosis infection, it is likely that neutrophils, rather than T-lymphocytes, are involved in inhibiting fungal spore development.

**References-**

1. Walther G, Wagner L, Kurzai O. 2019. Updates on the Taxonomy of Mucorales with an Emphasis on Clinically Important Taxa. J Fungi (Basel) 5.

2. Borman AM, Fraser M, Patterson Z, Palmer MD, Johnson EM. 2021. In Vitro Antifungal Drug Resistance Profiles of Clinically Relevant Members of the Mucorales (Mucoromycota) Especially with the Newer Triazoles. Journal of Fungi 7:271.

3. Çagatay AA, Öncü SS, Çalangu SS, Yildirmak TT, Özsüt HH, Eraksoy HH. 2001. Rhinocerebral mucormycosis treated with 32 gram liposomal amphotericin B and incomplete surgery: a case report. BMC Infectious Diseases 1:1-4.

4. Walsh TJ, Hiemenz JW, Seibel NL, Perfect JR, Horwith G, Lee L, Silber JL, DiNubile MJ, Reboli A, Bow E. 1998. Amphotericin B lipid complex for invasive fungal infections: analysis of safety and efficacy in 556 cases. Clinical Infectious Diseases 26:1383-1396.

5. Kofteridis D, Karabekios S, Panagiotides J, Bizakis J, Kyrmizakis D, Saridaki Z, Gikas A. 2003. Successful treatment of rhinocerebral mucormycosis with liposomal amphotericin B and surgery in two diabetic patients with renal dysfunction. Journal of chemotherapy 15:282-286.

6. Ericsson M, Anniko M, Gustafsson H, Hjalt C-Å, Stenling R, Tärnvik A. 1993. A Case of Chronic Progressive Rhinocerebral Mucormycosis Treated with Liposomal Amphotericin B and Surgery. Clinical Infectious Diseases 16:585-586.

7. Sun QN, Najvar LK, Bocanegra R, Loebenberg D, Graybill JR. 2002. In vivo activity of posaconazole against Mucor spp. in an immunosuppressed-mouse model. Antimicrob Agents Chemother 46:2310-2.

8. Heeres J, Meerpoel L, Lewi P. 2010. Conazoles. Molecules 15:4129-88.

9. Borman AM, Hughes JM, Oliver D, Fraser M, Sunderland J, Noel AR, Johnson EM. 2020. Lessons from isavuconazole therapeutic drug monitoring at a United Kingdom Reference Center. Medical mycology 58:996-999.

10. Bernardo V, Miles A, Fernandez AJ, Liverman R, Tippett A, Yildirim I. 2020. Initial posaconazole dosing to achieve therapeutic serum posaconazole concentrations among children, adolescents, and young adults receiving delayed-release tablet and intravenous posaconazole. Pediatr Transplant 24:e13777.

11. Durani U, Tosh PK, Barreto JN, Estes LL, Jannetto PJ, Tande AJ. 2015. Retrospective Comparison of Posaconazole Levels in Patients Taking the Delayed-Release Tablet versus the Oral Suspension. Antimicrob Agents Chemother 59:4914-8.

12. Petrikkos G, Skiada A, Lortholary O, Roilides E, Walsh TJ, Kontoyiannis DP. 2012. Epidemiology and clinical manifestations of mucormycosis. Clinical Infectious Diseases 54:S23-S34.

13. Sette A, Crotty S. 2021. Adaptive immunity to SARS-CoV-2 and COVID-19. Cell 184:861-880.

14. Grint DJ, Wing K, Williamson E, McDonald HI, Bhaskaran K, Evans D, Evans SJ, Walker AJ, Hickman G, Nightingale E, Schultze A, Rentsch CT, Bates C, Cockburn J, Curtis HJ, Morton CE, Bacon S, Davy S, Wong AY, Mehrkar A, Tomlinson L, Douglas IJ, Mathur R, Blomquist P, MacKenna B, Ingelsby P, Croker R, Parry J, Hester F, Harper S, DeVito NJ, Hulme W, Tazare J, Goldacre B, Smeeth L, Eggo RM. 2021. Case fatality risk of the SARS-CoV-2 variant of concern B.1.1.7 in England, 16 November to 5 February. Euro Surveill 26.

15. Ye Q, Wang B, Mao J. 2020. The pathogenesis and treatment of the `Cytokine Storm' in COVID-19. J Infect 80:607-613.

16. Giamarellos-Bourboulis EJ, Netea MG, Rovina N, Akinosoglou K, Antoniadou A, Antonakos N, Damoraki G, Gkavogianni T, Adami ME, Katsaounou P, Ntaganou M, Kyriakopoulou M, Dimopoulos G, Koutsodimitropoulos I, Velissaris D, Koufargyris P, Karageorgos A, Katrini K, Lekakis V, Lupse M, Kotsaki A, Renieris G, Theodoulou D, Panou V, Koukaki E, Koulouris N, Gogos C, Koutsoukou A. 2020. Complex Immune Dysregulation in COVID-19 Patients with Severe Respiratory Failure. Cell Host Microbe 27:992-1000 e3.

17. Chen G, Wu D, Guo W, Cao Y, Huang D, Wang H, Wang T, Zhang X, Chen H, Yu H, Zhang X, Zhang M, Wu S, Song J, Chen T, Han M, Li S, Luo X, Zhao J, Ning Q. 2020. Clinical and immunological features of severe and moderate coronavirus disease 2019. J Clin Invest 130:2620-2629.

18. Mcnulty JS. 1982. Rhinocerebral mucormycosis: Predisposing factors. The Laryngoscope 92:1140-1143.

19. Balachandar V, Mahalaxmi I, Subramaniam M, Kaavya J, Kumar NS, Laldinmawii G, Narayanasamy A, Reddy PJK, Sivaprakash P, Kanchana S. 2020. Follow-up studies in COVID-19 recovered patients-is it mandatory? Science of the Total Environment 729:139021.

20. Roilides E, Kontoyiannis DP, Walsh TJ. 2012. Host defenses against zygomycetes. Clin Infect Dis 54 Suppl 1:S61-6.

21. Artis WM, Fountain JA, Delcher HK, Jones HE. 1982. A mechanism of susceptibility to mucormycosis in diabetic ketoacidosis transferrin and iron availability. Diabetes 31:1109-1114.

22. Ibrahim AS, Spellberg B, Walsh TJ, Kontoyiannis DP. 2012. Pathogenesis of mucormycosis. Clinical Infectious Diseases 54:S16-S22.
